# Aprotinin—Drug against Respiratory Diseases

**DOI:** 10.3390/ijms241311173

**Published:** 2023-07-06

**Authors:** Alexandre V. Ivachtchenko, Andrey A. Ivashchenko, Dmitrii O. Shkil, Ilya A. Ivashchenko

**Affiliations:** 1 ChemDiv Inc., San Diego, CA 92130, USA; ai@chemdiv.com (A.A.I.); ilya.a.ivashchenko@gmail.com (I.A.I.); 2 ASAVI LLC, 1835 East Hallandale Blvd #442, Hallandale Beach, FL 33009, USA; dmitrii.shkil.medchem@gmail.com

**Keywords:** aprotinin, pan-protease inhibitor, serine protease inhibitor, influenza, SARS-CoV-2, COVID-19

## Abstract

Aprotinin (APR) was discovered in 1930. APR is an effective pan-protease inhibitor, a typical “magic shotgun”. Until 2007, APR was widely used as an antithrombotic and anti-inflammatory drug in cardiac and noncardiac surgeries for reduction of bleeding and thus limiting the need for blood transfusion. The ability of APR to inhibit proteolytic activation of some viruses leads to its use as an antiviral drug for the prevention and treatment of acute respiratory virus infections. However, due to incompetent interpretation of several clinical trials followed by incredible controversy in the literature, the usage of APR was nearly stopped for a decade worldwide. In 2015–2020, after re-analysis of these clinical trials’ data the restrictions in APR usage were lifted worldwide. This review discusses antiviral mechanisms of APR action and summarizes current knowledge and prospective regarding the use of APR treatment for diseases caused by RNA-containing viruses, including influenza and SARS-CoV-2 viruses, or as a part of combination antiviral treatment.

## 1. Introduction

Over 100 viruses can infect the cells of the respiratory tract and cause diseases in humans. The most frequent causative viruses of these respiratory diseases are adenoviruses, rhinoviruses, human coronaviruses, parainfluenza viruses, human metapneumovirus, respiratory syncytial virus hantavirus, influenza virus (IV), and severe acute respiratory syndrome (SARS) coronavirus (CoV) [1]. IV and CoV are among the most dangerous respiratory viruses causing pandemics. Thus, the Spanish influenza pandemic of 1918 resulted in the death of at least 50 million people worldwide [2], and the COVID-19 pandemic in 2019–2023 resulted in about seven million deaths [3]. Other estimates of the global number of deaths during the COVID-19 pandemic approaches 20 million [4,5,6].

There are four types of IVs: A, B, C, and D. Influenza A (IAV) and B (IBV) viruses cause seasonal epidemics in humans (known as “flu” season). Diseases range from mild to severe and even fatal. Worldwide, annual epidemics result in approximately three–five million cases of severe illness and approximately 290,000 to 650,000 deaths from respiratory diseases [7]. Only IAV cause pandemics in humans. Three influenza pandemics occurred in the 20th century: in 1918 (Spanish influenza, caused by A(H1N1), the deaths of 50–100 million people), 1957 (Asian influenza, caused by A(H2N2), the deaths of 1.1 million worldwide and 116,000 in the United States), and 1968 (Hong Kong influenza, caused by A(H3N2), the deaths of one million worldwide and about 100,000 in the United States). In addition, three influenza pseudo pandemics are known: in 1947 with low mortality rates, an epidemic in 1977 that was a pandemic among children, and swine influenza in 1976 that was feared to have pandemic potential [8]. In April 2009, a new pandemic A(H1N1)pdm09 virus appeared in Mexico and California, the United States, and was responsible for the first influenza pandemic of the 21st century [9]. CDC estimated that 151,700–575,400 people worldwide and 12,469 in the United States died from A(H1N1)pdm09 virus infection during the first year the virus circulated [10]. One of the candidates that could cause a pandemic in the future is a highly pathogenic avian A(H5N1) virus [11].

IBV cause a milder disease than some strains of IAVs, such as A(H3N2), but more active than A(H1N1) virus [12,13]. IBVs replicate, as do seasonal IAVs, in the cells of the upper respiratory tract, human bronchi, and occasionally in the lungs. IBVs infect ciliated, club, goblet, and basal cells in human airway organelles. Like seasonal IAVs, IBVs are low inducers of pro-inflammatory cytokines and chemokines. IBVs prefer the conductive airways over the lower lung [14]. In fact, numerous studies have shown increased activity of IBV in relation to the development of severe disease and mortality. IBVs have a significantly higher mortality rate in children compared to IAVs [15,16]. These data strongly refute claims that influenza B is a milder version of influenza. In humans, IBVs evolve slower than IAVs and faster than influenza C viruses (ICVs) [17,18].

Since the mid-1960s, seven CoVs were identified that can infect humans [19], four of them (OC43, HKU1, 229E, NL63) tend to cause mild symptoms. The other three human CoVs, MERS-CoV, SARS-CoV-1, and SARS-CoV-2, tend to cause severe respiratory syndromes [20]. SARS-CoV-1 [21] is a strain of CoV that causes SARS, the respiratory illness responsible for the 2002–2004 SARS outbreak [22]. MERS-CoV is a viral respiratory infection caused by Middle East Respiratory Syndrome related CoV first reported in Saudi Arabia in 2012 [23,24,25]. By July 2015, MERS-CoV cases had been reported in over 21 countries, in Europe, North America, and Asia as well as the Middle East (over 2600 cases) [26]. SARS-CoV and MERS-CoV are highly pathogenic CoVs that cause serious illness and about 10% and 36% mortality, respectively [27]. 

In December 2019 in Wuhan, China, the new SARS-CoV-2 outbreak was first reported and has turned out to be a global health emergency, causing COVID-19 to become another major RNA virus pandemic [3,28,29]. The COVID-19 pandemic ended only on 5 May 2023 [3] and has killed millions of people around the world and upended daily life in previously unimaginable ways [30]. In almost three years, SARS-CoV-2 has spread to 210 countries around the world. Globally, as of 3 May 2023, there have been 765,222,932 confirmed cases of COVID-19, including 6,921,614 deaths (0.9% mortality), reported to WHO [31].

**Figure 1:**
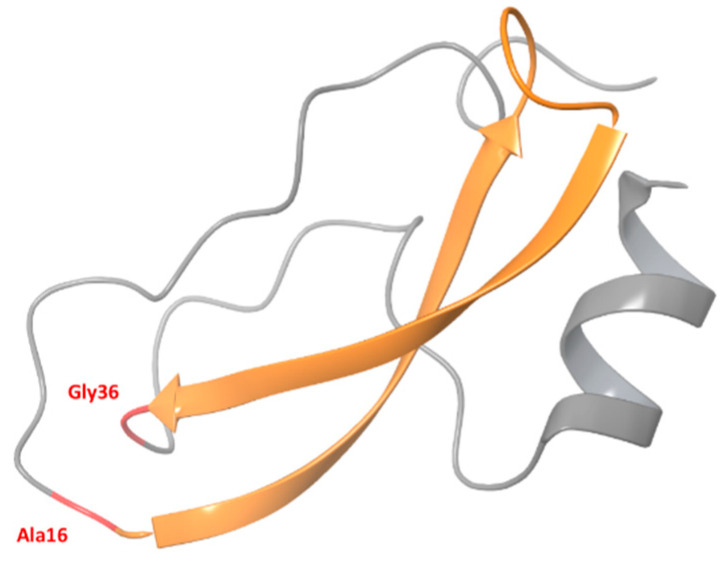
Tertiary structure of APR. The figure was constructed using X-ray data from RCSB PDB (PDB ID: 3LDJ). The molecule consists of a single polypeptide chain of 58 amino acid residues linked by three disulfide bridges. The molecule is about 29 Å long, 19 Å in diameter, and contains a double-stranded antiparallel β-sheet (from Ala-16 to Gly-36, orange color) twisted into a right-handed double helix with 14 residues per turn [56,57,58].

Development of optimal control measures against emerging viruses, especially those with pandemic potential, is an important goal. Vaccination is considered the best option for the control of viral diseases, but it would take 3–6 months to produce pandemic-matched, effective vaccines. Therefore, antiviral drugs can be an initial control measure. One of the promising candidates for the prevention and treatment of acute respiratory virus infections is aprotinin (APR) due to its ability to inhibit proteolytic activation of some viruses, and its activity against a broad range of viruses, including IVs and SARS-CoV-2. APR (Figure 1) was discovered in 1930 as an “inactivator” of kallikrein in bovine lymph nodes [1] and in 1936, as an inhibitor of bovine pancreatic trypsin [2]. It is also known as a bovine pancreatic trypsin inhibitor (BPTI) and a trypsin-kallikrein inhibitor (TKI). According to X-ray crystallography, BPTI has a three-dimensional pear-shaped molecule structure. The polypeptide chain is folded so that hydrophobic radicals are concentrated inside the molecule, while all hydrophilic radicals, with the exception of the side chain of Asp-43, are outside the molecule, exposed to the aqueous environment. This arrangement results in a very compact tertiary structure and is mainly responsible for the remarkable stability of APR against denaturation at high temperature, acids, alkalis, and organic solvents or proteolytic degradation. Other interesting features of APR are, on the one hand, its strongly dipolar character due to the concentration of negatively charged radicals at one end of the molecule, i.e., in the lower part of the pear, and on the other hand, its strong basicity molecules with an isoelectric point close to 10.5 [13,14,15]. It should be noted that, due to incompetent interpretation of several clinical trials to reduce perioperative blood loss and the need for blood transfusions in patients undergoing coronary artery bypass grafting with cardiopulmonary bypass [32,33,34,35,36,37], the usage of APR was nearly stopped for several years worldwide [38]. After incredible controversy in the literature [39,40,41,42,43,44] and re-analysis [38,41,42,43,44,45,46,47,48,49,50,51,52] of clinical trial data [32,33,34,35,36,37], restrictions on the use of APR were lifted worldwide in 2011–2020 [52,53,54,55]. This review discusses inhibitory activity and antiviral mechanisms of APR action and summarizes current knowledge and prospective regarding the use of APR prophylaxis and treatment for diseases caused by RNA-containing viruses, including IVs and SARS-CoV-2, or as a part of a combination antiviral treatment.

### 1.1. APR Inhibitory Activity

APR is a typical “magic shotgun” [59,60,61,62] pharmacological agent that reduces bleeding and limits the need for blood transfusion in cardiac and noncardiac surgeries [63,64,65]. It is also a promising drug in antiviral therapy [66,67] and especially in combination with other drugs [67,68].

APR is a competitive pan-protease inhibitor that forms loose complexes with serine proteases and blocks their active sites. It inhibits trypsin, chymotrypsin, and plasmin at a concentration of about 125,000 cfu/mL (KIU/mL) and kallikrein at a concentration of 300,000 cfu/mL (Table 1). Its action on kallikrein leads to inhibition of the formation of factor XIIa. As a result, both the internal coagulation pathway and fibrinolysis are inhibited. The action of APR on plasmin independently slows down fibrinolysis [69,70,71,72]. In addition, APR also inhibits the action of nitric oxide synthase types I and II and impairs K^+^ transport through Ca^2+^-activated K^+^ channels [73] and interacts with other factors of the coagulation and fibrinolytic cascade, creating a hemostatic balance without increasing the risk of thrombosis.

APR is an effective anti-inflammatory drug [74,75,76,77], which is called a “broad-spectrum anti-fibrinolysin” because of its anti-inflammatory and endothelial-modulating effects [78]. It has multiple actions that may suppress the inflammatory response, including attenuating platelet activation, maintaining platelet function, decreasing complement activation, inhibiting kallikrein production [79], decreased release of TNF-α [80], IL-6 and, IL-8 [81], inhibition of endogenous cytokine-induced iNOS induction [82], decreased CPB-induced leukocyte activation [79,83], and inhibition of up-regulation of monocyte and granulocyte adhesion molecules [84,85]. It may reduce lung injury, reduce bronchial inflammation [86], and attenuate reperfusion lung injury [87]. 

APR is an inhibitor of host serine proteases that cleaves the hemagglutinin (HA) glycoprotein of IVs and thus reduces the virus replication. In particular, it has been shown that plasmin cleavage of HA glycoprotein of IVs can be prevented by APR. The HA glycoprotein of IVs consists either of the precursor HA (75,000 kDa) or of its subunits HA1 (50,000 kDa) and HA2 (25,000 kDa). IVs cannot initiate infection of host cells unless the HA is proteolytically cleaved [88]. The HA1 and HA2 subunits are significantly more infectious than the HA precursor [89]. The HA glycoprotein of IVs plays a critical role in viral binding, fusion, and entry. That is why HA is an attractive target for inhibition of the initial stage of host cell infection with IVs. [90,91,92]. APR inhibits transmembrane protease serine S2 (TMPRSS2), which is essential in proteolytic activation of a broad range of viruses, including IVs and SARS-CoV-2 [93].

### 1.2. Adverse Effects of APR

The use of APR in major surgeries began in the 1960s, and the first publications addressing the anti-influenza activity of APR appeared in the early 1970s [94]. APR was generally well tolerated in patients undergoing surgery in clinical trials [95]. An important side effect with APR is hypersensitivity, including skin rashes, itching, dyspnea, nausea, tachycardia, and a fatal anaphylactic or anaphylactoid reaction (shock). The frequency of hypersensitivity reactions ranges from <0.1% to 5% [96,97,98,99]. Moreover, maximum hypersensitivity up to 5% was observed if patients were repeatedly exposed to APR within six months after initial administration [99].

Therefore, all intravenous doses of APR are administered through a central catheter. An initial (test) dose of 1 mL (10,000 KIU) APR is administered intravenously at least 10 min before the loading dose that is administered as a constant infusion dose (1M-2M KIU) [95]. 

An important side effect with APR is also a clinically significant but transient increase in serum creatinine and the potential for increased renal events. An increase in serum creatinine occurs in about 8% of patients and may persist for up to nine days. The mechanism is probably reuptake in the proximal tubules [100]. These effects are most common in patients with existing renal dysfunction [101].

## 2. Antiviral Treatment of Influenza

### 2.1. The Structure IAV, Function of Its Proteins, and HA Cleavage of IVs

**Figure 2:**
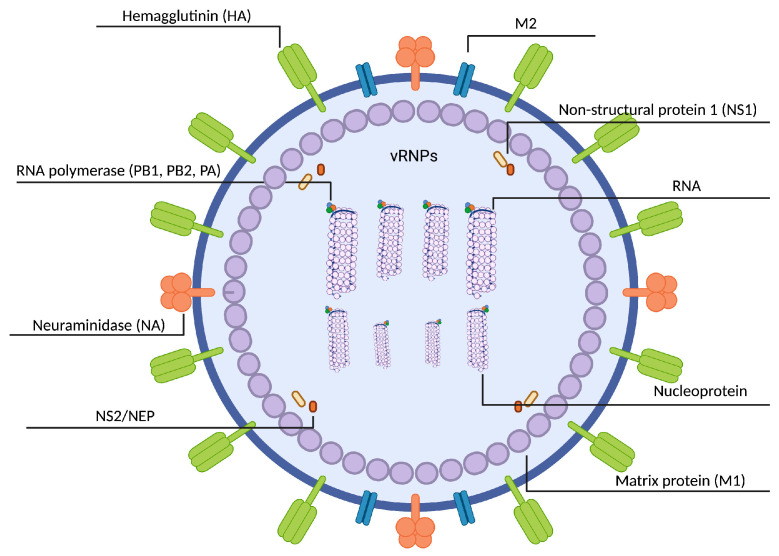
Schematic representation of the IAV. A lipid bilayer contains HA and NA glycoproteins and transmembrane ion channel M2 protein. M1 protein lies beneath the lipid bilayer and binds by NEP protein. Individual RNA segments are bound by a polymerase complex, consisting of the three proteins PA, PB1, and PB2, at their termini and encapsidated by the NP into a helical structure (RNP).

The IAV genome is a negative-sense, single-stranded, segmented RNA genome which is divided into eight segments that encode at least 11 viral proteins (Figure 2) [102]. The IAV is an enveloped virus consisting of an outer lipoprotein envelope and an inner ribonucleoprotein (RNP). The virus envelope contains four proteins: HA, neuraminidase (NA), the transmembrane ion channel matrix 2 (M2), and a small amount of the nuclear export protein (NEP). RNP contains RNA and four polypeptides: the main nucleocapsid protein (NP), polymerase basic 2 (PB2), polymerase basic 1 (PB1), and polymerase acidic (PA). Both modules are connected to each other by the matrix 1 (M1) protein, which maintains viral integrity [103].

The HA attaches virions to sialic acid (SA) fragments of host receptors; NA is not required for viral replication but required for budding of newly formed viral particles from the surface of infected cells. It facilitates virus movement to the target cell by cleavage of SA receptors from respiratory tract mucins, and helps the release of virions from infected cells; the M1 protein is a membrane-binding and RNA-binding protein and forms a coat inside the viral envelope, determines the virion’s shape, interacts with vRNP and other cytoplasmic domains of integral membrane proteins, increases vRNPs export and decreases import, and helps assembly and budding of virions; the M2 protein is vital for viral replication, forms a proton channel in the virus envelope, lowers the pH inside the viral particle to promote uncoating of RNPs, modulates Golgi’s pH, and helps to stabilize HA’s native conformation during virus assembly; the nonstructural protein 1 (NS1) acts as a promoter of viral replication and an inhibitor of the host's immune response; NP binds nonspecifically to single-stranded RNA (ssRNA), encapsidates viral RNA, and helps recruiting RNA polymerase for synthesis of viral positive-sense RNA (cRNA); the NS2/NEP protein promotes viral RNA replication, regulates vRNP’s export from the nucleus to the cytoplasm, RNA nuclear export, and interacts with the viral M1 protein; the PA has presumably helicase-like functions and is important for viral transcription and assembly of the polymerase complex; the main PB1 responsible for elongation of the primed nascent viral mRNA is located in the nucleus of infected cells, enhances the association of three subunits of the RNA polymerase complex; the PB2, located in the nucleus of infected cells, signals the viral polymerase passage to the host’s nucleus, enhances the formation of the cap structures necessary for viral messenger RNA (mRNA) transcription, located in the mitochondria of infected cells, inhibits Interferon-β (IFN-β), and helps determine host range [104]; the PB1-F2 protein contributes to viral pathogenicity [105]. Virus entry into the host cell, replication, assembly, and movement of the IVs virions are detailed in the reviews [106,107,108].

The TMPRSS2 is expressed in epithelial cells of the human respiratory tract and cleaves (activates) the HA glycoprotein of IAVs and IBVs into HA1 and HA2 subunits to allow virus fusion with host cell receptors [109,110,111]. It was first identified in 2006 by Bottcher and colleagues [109]. Along with TMPRSS2 [109], host proteases with trypsin-like activity, such as TMPRSS4, TMPRSS11D, ST14, KLK5, KLK12, TMPRSS11E, and TMPRSS13, have also been shown to cleave HA glycoprotein of IAVs and IBVs and support viral replication in cell cultures [111,112,113,114,115,116]. At the same time, the proteases prostasin, hepsin, TMPRSS3, TMPRSS6, TMPRSS9, TMPRSS10, TMPRSS11B, and TMPRSS11F do not activate HA glycoprotein of IAVs and IBVs when co-expressed in mammalian cells [114,117,118,119]. 

Mice deficient in TMPRSS2 expression were protected from lethal challenge with A(H7N9) or A(H1N1)pdm09 viruses but were resistant to challenge with A(H3N2) virus. This suggests that activation of HA glycoprotein of A(H3N2) virus is independent of TMPRSS2 [120,121,122,123]. 

TMPRSS2 was found to be crucial for proteolytic activation of the avian IAVs of H1-H11, H14, and H15 subtypes in human and mouse airway cells [124]. Only H9 (with a R-S-S-R cleavage site) and H16 avian IAVs were proteolytically activated in the absence of TMPRSS2 activity, albeit with reduced efficiency. It was also shown that in human and murine airway cells, TMPRSS2 is the major activating protease of IAV of almost all HA subtypes having a monobasic HA cleavage site. An additional exception was HA of IBVs in human and mouse respiratory cells, which did not depend on TMPRSS2 activation [123].

Proteolytic cleavage regulates numerous processes in human metabolism and immune responses. One key player is the ubiquitously expressed serine protease furin, which cleaves a plethora of proteins at polybasic recognition motifs. Mammalian substrates of furin include cytokines, hormones, growth factors, and receptors [125]. Generally, HA of human and low pathogenic avian IAVs cannot be cleaved by furin as they usually only harbor a mono- or dibasic HA cleavage site. Instead, they depend on trypsin-like proteases such as TMPRSS2 or human airway trypsin-like protease for activation [109]. Expression of such trypsin-like proteases is largely restricted to the respiratory and gastrointestinal tract. In contrast, HA of many highly pathogenic avian influenza A(H5N1) and A(H7N9) viruses can be cleaved by furin or PCSK5, which are present in many cell types [126]. Thus, the ability to exploit furin for efficient HA cleavage and the associated increase in pathogenicity are determined by the presence of a furin consensus target site, but also by adjacent residues and the absence of masking oligosaccharide chains.

### 2.2. Antiviral Drugs Available for Influenza Treatment

Influenza affects about 3% to 10% of the world’s population annually. The most common complications of influenza include viral [127] or bacterial co-infections [128,129], which lead to the death of about half a million people each year [130]. CDC estimates that influenza caused 29–41 million illnesses, 380,000–710,000 hospitalizations, and 22,000–38,000 deaths annually between 2010 and 2020 [131,132]. 

During the influenza season in the United States, mortality attributed to influenza associated with pneumonia ranges from 5.6% to 11.1% [133]. In a cohort study including laboratory-confirmed influenza cases, those admitted with pneumonia, were more likely to require admission to an intensive care unit (ICU, 27% vs. 10%), mechanical ventilation (18% vs. 5%), and higher risk of death (9% vs. 2%) [134]. In 2020, the CDC ranked influenza complicated with pneumonia as the ninth leading cause of death in the United States [135]. 

Vaccination is considered the most effective strategy for preventing and controlling influenza in humans [7,130]. However, current influenza vaccines have several limitations, including their limited efficacy when there is an antigenic mismatch between the vaccine composition and circulating viruses [7]. The effectiveness of seasonal prophylaxis with influenza vaccines developed over several decades ranges from 10% to 60% [135,136,137].

There are currently seven viral target proteins, including nine antivirals approved for the treatment of influenza (Table 2) [138,139,140,141,142,143,144,145].

Historically, treatment options for influenza infections were limited to four classes of virus protein-specific drugs targeting M2, NA, PB1, or PA proteins. The first of them, inhibitors of ion channel activity of M2 protein (amantadine, rimantadine) are active only against IAV [146]. M2 inhibitors have lost their relevance because IAVs are resistant to these compounds [147,148,149] and IBVs are insensitive to M2 inhibitors [138,148,150,151]. 

The NA inhibitors (NAI) are targeting the surface of NA glycoprotein (oseltamivir, zanamivir, peramivir, and laninamivir), and act against IAV, IBV, and ICV. The prevalence of viruses resistant to NAIs in global circulation is generally low (<2.0%) [152]. However, with only a single oral-dosed NAI oseltamivir available on the market, the development of new and improved anti-influenza drugs is important [152,153,154]. The drug candidate effective against oseltamivir and zanamivir resistant viruses is the NAI AV5080 [155,156]. 

Favipiravir (FVP) is an inhibitor of the RNA-dependent RNA polymerase (RdRp) of a broad range of RNA viruses, and it inhibits viral RNA synthesis as a chain terminator [157]. Emerging IV, with antigenically distinct surface glycoproteins and composition of internal genes than seasonal IV, cause severe disease and high mortality rates—53.5% for A(H5N1) [158] and 34% for A(H7N9) virus infections [159]. FVP demonstrated antiviral activity against different subtypes of IV in animal models, including highly pathogenic A(H5N1) and oseltamivir resistant viruses [160,161,162,163]. FVP was more efficacious than oseltamivir in inhibiting replication of the A/Puerto Rico/8/1934 (H1N1) virus in vitro and the protection of mice infected with a high dose of this virus [164]. Following clinical trials, FVP was approved for restricted use and pandemic stockpiling in Japan in 2014 [165]. The results of two randomized, double-blind, placebo-controlled phase 3 (US316 and US317) international trials of FVP treatment of uncomplicated influenza in adults have recently been published. US316 (NCT02026349) was conducted in 14 countries in Africa, Europe, Asia, Australia and New Zealand, and the United States over three influenza seasons between January 2014 and March 2015. US317 (NCT02008344) was conducted in 10 countries and territories in the Americas between December 2013 and February 2015. In both studies, FVP demonstrated a decrease in viral titers within 1–5 days after initiation of treatment and the median time to loss of virus detection decreased by 23.2–24.0 h compared with placebo (P < 0.001). Adverse events were generally mild or moderate. The authors recommended to conduct additional studies and investigate higher FVP doses and drug combinations for the treatment of severe influenza and other RNA-containing viral infections [166].

Baloxavir marboxil (BXM) is a prodrug of the biologically active baloxavir acid (BXA) and inhibits cap-dependent endonuclease (CEN) activity of PA proteins of IAV, IBV and ICV. BXM is the first inhibitor of this type approved in Japan (2018), the United States (2018), and Europe (2021) [167,168,169]. A significant advantage of BXM over NAIs is its weight-adjusted single oral dose administration regimen [170,171]. The emergence of IV with PA-I38T substitution was already detected on day three after treatment with BXM (range three–nine days), and in most cases this occurred on day five and may lead to virus rebound [172,173,174]. The BXM analog AV5124 (prodrug of AV5116) exhibited low cytotoxicity in MDCK cells and lacked mitochondrial toxicity, resulting in favorable selective indexes. AV5116 was equipotent or more potent in vitro than BXA against wild-type viruses and viruses with reduced BXA susceptibility carrying a PA-I38T substitution [175]. AV5124 showed promising efficacy as an anti-influenza drug candidate in a mouse animal model [144,175,176], and treatment with 20 mg/kg or 50 mg/kg prevented death in 60% and 100% of animals, respectively [176]. 

Host-targeted antiviral drugs, such as inhibitors of proteolytic activation of the HA glycoprotein of IVs (serine protease inhibitors), only recently appeared in influenza therapy. This group includes camostat, nafamostat [177], and APR [178]. However, in contrast to the efficacy in vitro [179], the clinical efficacy of camostat and nafamostat is still unclear. At the same time, a hand-held metered-dose inhaler containing APR (Aerus^TM^) for the treatment of influenza was developed and is used in Russia [109,180,181].

### 2.3. APR for Influenza Treatment

In our opinion, the main advantages of APR over existing/proven antiviral drugs are its TMPRSS2 activity, thus inhibiting virus entry into host cells and virus replication [112,118,119,182,183]. It is an excellent partner for combination therapy because it has a different mechanism of anti-influenza action than existing/approved antiviral drugs. In addition, it inhibits the processes of inflammation [63,74,75,76,77] and vascular thrombosis [70,71,72,73], which are very important in the treatment of complications (particularly pneumonia) caused by influenza infection.

The first publications reporting the anti-influenza activity of APR appeared in the early 1970s. It was shown that cleavage of the HA glycoprotein of IV by plasmin can be prevented by Kunitz trypsin inhibitors from bovine pancreas, i.e., by APR [94]. APR has been actively studied as an anti-influenza drug since the early 1980s. It should be noted that a significant contribution to these studies was made by Zhirnov and colleagues [89,178,179,184,185,186,187,188]. They demonstrated APR activity against a number of IAVs: A/Puerto Rico/8/1934 (H1N1) [89,178], A/Aichi/2/1968 (H3N2) [89,188], A/California/04/2009 (H1N1)pdm09 [178], A/Hamburg/05/2009 (H1N1)pdm09 [178], and oseltamivirresistant A/Brisbane/10/2007 (H3N2) [189]; and IBVs: B/Hong Kong/1973 [187,190] and B/Lee/1940 [187,190]. 

More recently, Song et al. [145] investigated antiviral APR activity in vitro among different IAV subtypes, including seasonal human IAVs [A/Puerto Rico/8/1934 (H1N1), A/California/04/2009 (H1N1)pdm09, A/Philippines/2/1982 (H3N2), A/Brisbane/10/2007 (H3N2)], avian IAVs [A/aquatic bird/Korea/CN2/2009 (H5N2), A/aquatic bird/Korea/CN5/2009 (H6N5), A/chicken /Korea/01310/2001 (H9N2)], and oseltamivirresistant IAV: [A/Brisbane/10/2007 (H3N2)] and IBV [B/Seoul/32/2011 (Yamagata-like lineage)]. The APR EC_50_ values against different IAV ranged from 11 nM to 110 nM and were 39 nM for IBV [191]. The anti-influenza activity of APR was confirmed in mice lethally challenged with A/Puerto Rico/8/1934 (H1N1) virus [191].

In 2011, Zhirnov et al. suggested an aprotinin-based aerosol preparation for treatment of respiratory viral infections [192]. Zhirnov et al. showed the efficacy of aerosol formulation of APR for the treatment of experimental influenza and parainfluenza bronchopneumonia in mice [109,180,181,193,194,195,196,197,198]. In humans, APR aerosol demonstrated efficacy against natural influenza and parainfluenza infections when administered by inhalation using a manual aerosol inhaler Aerus^®^ [109,181]. The study was conducted in Russia during the winter–spring outbreak of influenza caused by the pandemic A(H1N1)pdm09 virus. Patients inhaled two aerosol doses of APR (160 KIU) three times a day for four–five days. In the comparison group, patients received a single oral dose of Ingavirin™ (90 mg) for five days. The authors found an approximately 10-fold reduction in viral load in patients treated with APR compared to those treated with Ingavirin™. The duration of clinical symptoms such as rhinorrhea, weakness, headache, sore throat, cough, chest pain, and fever was one–two days shorter in the APR-treated group than in the Ingavirin™-treated group. Side effects and discomfort in patients of the APR group were not detected [192].

Acute myocarditis is a well-known complication of influenza infection and a common prelude to inflammatory dilated cardiomyopathy (DCM) that can lead to chronic heart failure [181,199]. IAV-induced trypsin expression in the myocardium triggers acute viral myocarditis through stimulation of IAV replication, pro-MMP-9 activation, and cytokine induction. It was reported that inhibition of trypsin can prevent DCM with improvement in cardiac function after A/Puerto Rico/8/1934 (H1N1) virus infection of mice [200,201,202]. It was shown that ectopic myocardial trypsin was involved in acute and chronic myocardial inflammation, promoting IAV infection and initiating the trypsin-MMP-9 cytokine cycle, and promoting progressive cardiac dilatation through collagen remodeling. Trypsin plays an important role in the development of DCM after IAV infection, and APR prevents the progression of myocarditis to DCM by suppressing IAV infection, interrupting the trypsin-MMP-9-cytokine cycle, and restoring collagen metabolism through inhibition of trypsin activity. Thus, pharmacological inhibition of trypsin activity may be a promising approach to the prevention of virus-induced cardiomyopathy. To date, no clinical data on the use of APR for the prevention of inflammatory DCM are available.

In conclusion, it should be noted that the efficacy of APR for the treatment of influenza and acute respiratory diseases (ARD) has strong experimental evidence in vitro and in animal models. However, there are no clinical trial data on the treatment of complications of influenza or ARD, such as pneumonia, either with APR or with combination therapy that includes APR and an anti-influenza drug with a mechanism of action other than APR. Given the pan-protease activity of APR and rehabilitation of APR, the time has come for the conclusive clinical trials using APR for the treatment of influenza pneumonia.

## 3. APR for COVID-19 Treatment

### 3.1. SARS-CoV-2

**Figure 3:**
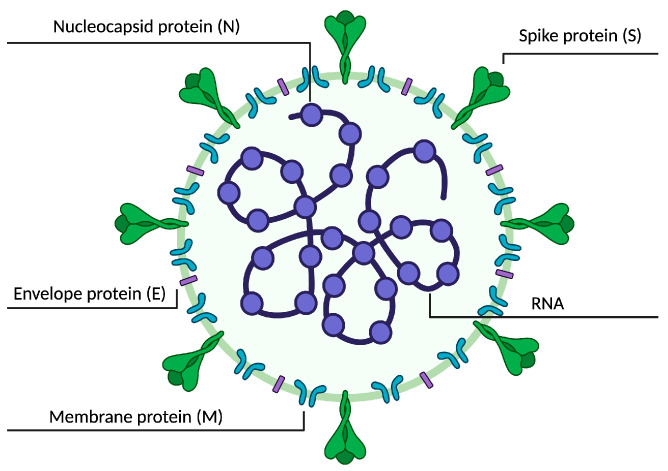
Schematic representation of the SARS-CoV-2 virus. A lipid bilayer contains S protein, the M glycoprotein and E protein cloaks the helical nucleocapsid, which consists of the N protein that is associated with the viral RNA. The lipid envelope is derived from intracellular membranes [203].

The typical CoV genome is a single-stranded, nonsegmented RNA genome, which is approximately 26–32 kb. It contains 5′-methylated caps and 3′-polyadenylated tails and is arranged in the order of 5′, replicase genes, and genes encoding structural proteins: spike (S) glycoprotein, which exists as a homotrimer and forms the characteristic spikes found on the surface of the virus. Acting as a fusion protein, it allows the virus to enter the host cell after being recognized by the angiotensin-converting enzyme 2 (ACE2) receptor; envelope (E) protein, which forms the envelope of the virus; membrane (M) protein, which forms a matrix that connects the envelope with the inner part of the virus; and nucleocapsid (N) protein, which holds the viral genome, part of the positive RNA strand (Figure 3). 

The partially overlapping 5′-terminal open reading frame 1a/b (ORF1a/b) is within the 5′ two-thirds of the CoV genome and encodes the large replicase polyprotein 1a (pp1a) and pp1ab. These polyproteins are cleaved by papain-like cysteine protease (PLpro) and 3C-like serine protease (3CLpro) to produce nonstructural proteins, including RdRp and helicase (Hel), which are important enzymes involved in the transcription and replication of CoVs. The 3′ one-third of the CoV genome encodes the structural (S, E, M, and N) proteins, which are essential for virus–cell receptor binding and virion assembly, and other nonstructural and accessory proteins that may have immunomodulatory effects [204,205,206,207,208,209].

The entry steps of the viral particles—encompassing attachment to the host cell membrane and fusion—are mediated by the S protein, which is assembled as a homotrimer and is inserted in multiple copies into the membrane of the virion giving it its crown-like appearance. The mechanism of penetration of SARS-CoV-2 into the host cell, in particular, includes the binding of the virus S-protein to the ACE2 receptor of the host cell and subsequent fusion of their membranes [210]. The cellular protease furin cleaves the S protein at the S1/S2 site and this cleavage is essential for S-protein-mediated cell–cell fusion and entry into human lung cells. In this regard, furin can be considered as a potential target for therapeutic intervention [211,212,213].

As TMPRSS2 is present at the cell surface, TMPRSS2-mediated S protein activation occurs at the plasma membrane, whereas cathepsin-mediated activation occurs in the endolysosome. Thus, the S protein on the mature virion consists of two noncovalently associated subunits: the S1 subunit binds ACE2 and the S2 subunit anchors the S protein to the membrane. The S2 subunit also includes a fusion peptide and other machinery necessary to mediate membrane fusion upon infection of a new cell [214].

How SARS-CoV-2 crosses the airway barrier of mucus and periciliary mucins to infect the nasal epithelium remains unclear. Recently, Wu et al., using primary cultures of nasal epithelial organoids, found that the virus attaches to motile cilia via the ACE2 receptor [215]. SARS-CoV-2 traverses the mucosal layer using motile cilia as pathways to access the cell. Cilia depletion blocks infection by SARS-CoV-2 and other respiratory viruses. SARS-CoV-2 progeny attach to airway microvilli 24 h after infection and induce the formation of apically elongated and highly branched microvilli, which organize the release of the virus from the microvilli back into the mucus layer, supporting the model of virus spread through the airway tissue via mucociliary transport. Importantly, Omicron variants bind with higher affinity for motile cilia and exhibit accelerated viral entry. Motile cilia, microvilli, and mucociliary-dependent mucus flow are hypothesized to be critical for efficient viral replication in the nasal epithelium [215].

Recent genetic changes in SARS-CoV-2 have increased both transmissibility of the virus and hospitalization rates [216,217]. A positive relationship between transmissibility and hospitalizations may reflect a common underlying mechanism: higher affinity of SARS-CoV-2 for ACE2 can increase both. More efficient binding to ACE2 can support replication in the upper respiratory tract, promoting more efficient transmission, and it can also increase replication in the lower respiratory tract and systemically, causing a severer disease. One outstanding question is whether the S protein has reached the maximum affinity for human ACE2 through changes in the receptor-binding domain (RBD) or whether it will further mutate and continue to enhance both transmissibility and pathogenicity [217]. 

SARS-CoV-2 has undergone many changes in the last three years, and some genetic changes result in higher virulence and/or transmission potential. The Delta variant penetrates lung cells more easily than the Wuhan-like virus that circulated in the early stages of the pandemic and is more effective in syncytia formation of infected and uninfected lung cells. This contributes to the more severe progression of COVID-19. The Delta variant is believed to be more than twice as infectious compared to previous SARS-CoV-2 variants [211]. 

The new variant of SARS-CoV-2 called Omicron has caused global panic and concern owing to its contagious and vaccine-escape substitutions. Presently, up to 60 substitutions have been identified in the BA.1 lineage, with as many as 38 of these occurring in the S protein, one in the E protein, two in the M protein, and six in the N protein. BA.2 lineage possesses 57 substitutions, with 31 in the S protein, of which the N-terminus is significantly different from that of BA.1 [218]. The RBD of the S protein is responsible for binding to the host receptor ACE2 and has the potential to increase infectivity and mediate escape from vaccine-induced neutralizing antibodies [219,220,221]. All current vaccines based on the original Wuhan strain provide weak protection against Omicron [222]. 

The hazard ratio (HR) for hospitalization or death among Omicron cases compared with Delta cases was 0.41, while the HR for ICU admission or death was 0.19, and the HR for death was 0.12. Stratified estimates of Omicron severity by age, sex, and vaccination status all indicated reduced Omicron severity [223]. A subvariant of Omicron, XBB.1.5 (Kraken), is the most transmissible strain of the SARS-CoV-2 and is from the XBB family of variants that emerged a few months ago and caught virologists’ attention because it contains more substitutions to evade immunity than other variants seen so far. The XBB.1.5 subvariant has a substitution that is believed to help the virus bind to cells, becoming more transmissible [224].

The WHO-declared end of the COVID-19 public health emergency in May 2023 should be interpreted with caution. Current evidence indicates that the efficacy of a fourth dose of classical mRNA vaccines (BT162b2 or mRNA-1273) is low and short-lived in preventing SARS-CoV-2 infection in its predominant Omicron variant [225].

It is believed that the Omicron variant is probably not the last variant, its “effectiveness” is expected to decrease as immunity increases in the population due to vaccines and infections. Although no new genetic changes have been discovered until recently, the general trend is that the virus is becoming less dangerous, mainly due to enhanced immunity in the population worldwide [226]. Omicron spikes do not efficiently use TMPRSS2 to enter cells, but rely mainly on the endocytic pathway, resulting in reduced replication in the lung parenchyma and an increased ability to infect the upper respiratory tract, making the virus less pathogenic. Omicron has a reduced ability to induce syncytia in tissue culture, which is potentially of clinical relevance as syncytia formation is associated with increased disease severity. Syncytium formation usually requires viral infection via membrane fusion involving TMPRSS2. The low rate of syncytia formation upon Omicron infection suggests that it instead switches to using endosomal fusion via cathepsins. Routine vaccination or previous infection cannot provide effective protection against Omicron, so revaccination is required. In addition, only a few pharma-developed neutralizing monoclonal antibodies are active against Omicron, while most antivirals in development are effective against it [226,227,228,229].

The replication of the viral genome within the infected cells is a key stage of the SARS-CoV-2 life cycle. It is a complex process involving the action of several viral and host proteins to perform RNA polymerization, proofreading, and final capping. Understanding the molecular mechanisms that guide the replication of this coronavirus is essential to develop therapeutic tools to neutralize SARS-CoV-2 [230].

### 3.2. Emergency Use of Inhibitors for COVID-19 Treatment

For drug development, the best-known targets of SARS-CoV-2 are the S protein, the main protease Mpro (also called 3CLpro and nsp5), the RdRp [231], as well as the main host protease TMPRSS2 and furin, which mediates the entry of SARS-CoV-2 into host cells by priming the S protein [93,232]. Since the announcement of the COVID-19 pandemic, there has been a tremendous amount of research into drug development against SARS-CoV-2, usually based on the repurposing of known antiviral compounds and drugs [231]. For example, until March 2021, 4952 clinical trials have been registered in ClinicalTrials.gov toward the drug and vaccine development for COVID-19. More than 100 countries have participated in contributing to these clinical trials [233].

**Figure 4:**
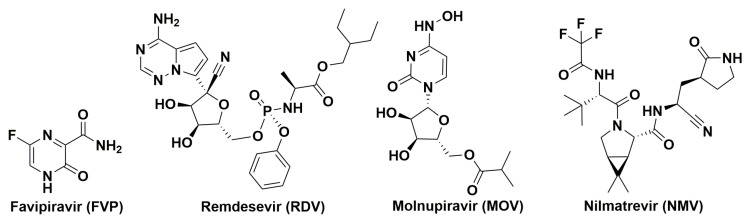
Chemical structures of antiviral drugs approved for the emergency use for treatment of COVID-19 patients.

As a result of these efforts, only a few anti-SARS-CoV-2 compounds have been identified targeting RdRp and Mpro (Figure 4). In different cell types, it was shown that Omicron viruses remain sensitive to FVP, remdesivir (RDV), molnupiravir (MOV), and nilmatrelvir (NMV) [234,235,236]. These drugs are inhibitors of SARS-CoV-2 replication in the host cells [237,238,239,240]; however, their efficacy in the treatment of COVID-19 patients has been debated to date [189,241,242,243,244]. FVP selectively and effectively inhibits the RdRp of RNA viruses [245,246]. FVP proved to be the first oral anti-SARS-CoV-2 drug, that effectively reduces viral clearance within seven days and contributes to clinical improvement within 14 days.

RDV (GS-5734, Veklury) is an RNA polymerase inhibitor of SARS-CoV-2 [247]. On October 22, 2020, the FDA approved RDV for the treatment of patients hospitalized with COVID-19 [248]. At the time, WHO-launched the global “Solidarity” trial comparing four treatment options for COVID-19 and found that RDV did not have a substantial effect on patients’ length of hospital stay or chances of survival [249,250,251,252,253,254]. In this regard, on 20 November 2020, WHO issued a conditional recommendation against the use of RDV in hospitalized patients, regardless of the severity of the disease [254]. However, RDV has been approved for emergency use for COVID-19 in many countries [255], including for intravenous administration in adults and pediatric patients [256]. Note that the first publication of the negative results of the “Solidarity” trial was published five months before the FDA decision [250].

MOV is an RNA polymerase inhibitor of SARS-CoV-2 [257] and other RNA-containing viruses such as Ivs and Ebola. MOV received its first approval on 4 November 2021 in the UK for the treatment of mild-to-moderate COVID-19 in adults with a positive SARS-CoV-2 diagnostic test and who have at least one risk factor for developing severe illness [258,259]. On 23 December 2021, based on several clinical trials [260,261,262,263,264,265], the FDA approved the emergency use of MOV for the treatment of patients with mild-to-moderate COVID-19 [266]. On 3 March 2022, WHO updated its living guidelines on COVID-19 therapeutics to include a conditional recommendation on MOV which should be provided only to nonsevere COVID-19 patients with the highest risk of hospitalization [267].

NMV is an inhibitor of Mpro, which is also referred to as 3CLpro or nsp5 protease inhibitor [241,268]. NMV, in combination with PaxlovidTM and Ritonavir (RTV), received its first emergency use authorization from the European Medicines Agency on December 16, 2021 [269]. PaxlovidTM was approved for the treatment of adults with mild-to-moderate COVID-19 in Canada on 17 January 2022, and later received a conditional marketing authorization from the European Commission on 27 January 2022 [270]. A low dose of RTV is included in PaxlovidTM to slow the breakdown of NMV, allowing it to stay longer in the body to fight COVID-19 [271]. The combination of NMV + MOV showed significant synergy in tissue culture [237], in mouse [272] and rhesus monkey [273] models compared with the effectiveness of the monotherapies. Clinical trials [274,275] have confirmed the effectiveness of the NMV + MOV combination against the Omicron variant.

Several drug combinations for the treatment of COVID-19 patients were studied, such as FVP + hydroxychloroquine (HCQ) [276,277], FVP + methylprednisolone [278,279], FVP + lopinavir-ritonavir [280], FVP + camostat + ciclesonide [281], RDV + Olumiant^®^ [282], RDV + Tocilizumab [283,284,285], RDV + LY-CoV555 [286], RDV + IFN β-1a [287], RDV + dexamethasone [288], MOV + IFN-α (in Calu-3 cells) [289], and MOV + FVP (in hamster infection model) [290]. Of the drug combinations listed above, only the combination of RDV + Olumiant^®^ was found to be more effective than RDV monotherapy [291]. On 19 November 2020, the FDA issued an emergency use authorization for RDV + Olumiant combination for the treatment of certain categories of hospitalized patients [291].

### 3.3. Entry Inhibitors of SARS-CoV-2

Among the various stages in the virus life cycle, its entry into the host cell is the most attractive therapeutic target for drug development. Designing inhibitory drugs that can interfere with the virus entry process constitutes one of the main preventative therapies that could combat SARS-CoV-2 infection at an early stage [292]. Of greatest interest were apparently synthetic serine protease inhibitors including nafamostat mesylate [293] and camostat mesylate [294]. However, clinical trials of these drugs have not shown clinical benefit in patients with mild-to-moderate COVID-19 [295,296,297], and hospitalized patients [298,299].

To date, despite intensive screening and drug discovery efforts, no SARS-CoV-2 entry inhibitor has reached clinical use; however, their development continues. In our opinion, APR is an excellent drug candidate for the prevention and treatment of COVID-19 [300]. Unlike known SARS-CoV-2 inhibitors approved for emergency use for the treatment of COVID-19 infection, APR is an inhibitor of SARS-CoV-2 entry into the host cell [301]. It is also an inhibitor of SARS-CoV-2 induced thrombo-inflammation, IFN-α release, expression of granulocyte and emomonocyte adhesion molecules, nitric oxide synthase (NOS), tracheobronchial secretion and plasminogen, and preventing activation of complement proteins C3a and C5a, which cause acute inflammation [58,74,302]. APR inhibits activation of mast cells, neutrophils, and endothelial cells [58,74,302]. APR has an antithrombotic mechanism of action, which, in combination with its multilevel anti-inflammatory activity, makes this drug a valuable assistant in COVID-19 treatment. APR can be used alone for the prevention and treatment of COVID-19 [59,66] or in combination with inhibitors of SARS-CoV-2 replication in the host cells [67,68,303,304].

#### 3.3.1. APR for COVID-19 Prevention

**Figure 5:**
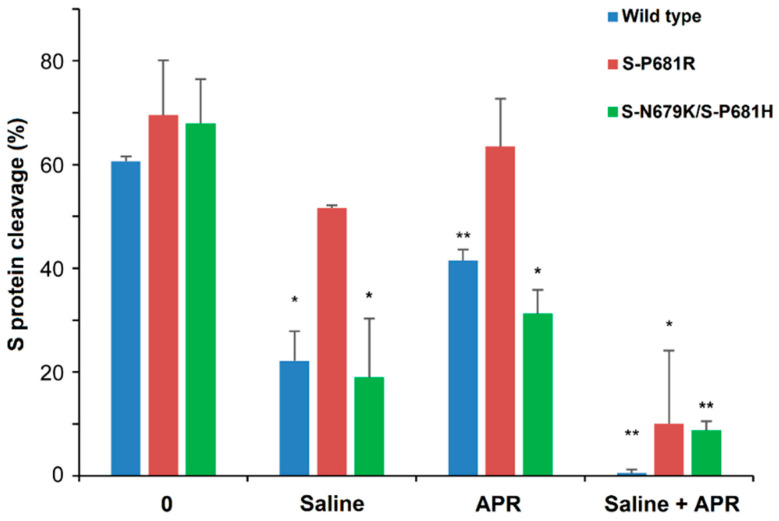
Inhibition of S protein cleavage of the SARS-CoV-2 wild-type, S-P681R, and S-N679K/S-P681H mutants caused by administration of hypertonic saline alone (3%); APR (2 µg/well); hypertonic saline, and APR combination (3% + 2 µg/well). * *p* < 0.05; ** *p* < 0.01 [305].

It has recently been shown that a solution of APR in saline effectively blocked the furin site cleavage in both the SARS-CoV-2 wild-type and mutants (P681R and N679K/P681H). APR reduces 99% of furin cleavage in the Wuhan-like SARS-CoV-2, 90% in the Omicron variant with S-P681R substitution, and 83% in the Omicron variant with S-N679K/S-P681H substitutions (Figure 5). This could represent a simple, economical, and practical feasible approach in locally controlling viral activation and entry into cells to replicate, i.e., a method of prevention of the SARS-CoV-2 infection. 

Previously, a nonpeer-reviewed preprint was published on the COVID19-PREPRINTS.MICROBE.RU platform, which shows the preventive efficacy of aprotinin in the model of Syrian hamsters infected with SARS-CoV-2 and in medical workers constantly working in the “red zone” of a COVID-19 hospital [66].

**Figure 6:**
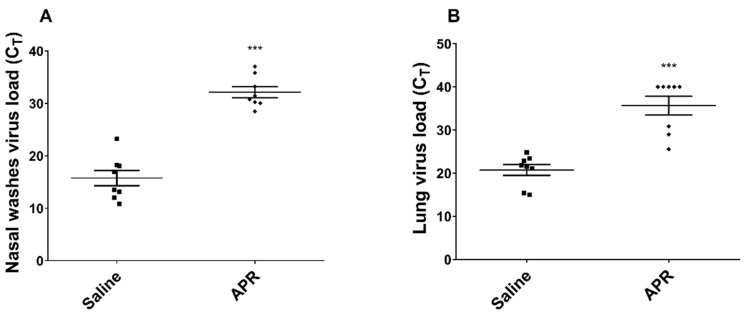
Efficacy of APR prophylaxis on SARS-CoV-2 virus load nasal washes (**A**) and in lungs (**B**) of infected hamsters. Hamsters (n = 8/group) were lightly anesthetized, and APR (1000 KIU/animal) was administered intranasally twice daily for three days. The control (virus-inoculated, untreated) animals received saline on the same schedule. One hour after the first APR dose, each hamster was inoculated with 10^3^ CPE_50_ of SARS-CoV-2 (100 µL/animal). Virus load was determined by qRT-PCR (limit of detection, CT = 40) at three days after infection. *** *p* < 0.001 compared to control virus-inoculated untreated animals (unpaired *t*-test) [66].

Syrian hamsters were randomized into two groups: APR-treated (500 KIU/nostril, 1000 KIU/animal) and control PBS-treated. Animals were challenged with SARS-CoV-2 at a dose of 1000×CPE50/animal 1 h after drug treatment. Animals were treated three times a day and continued for two days. Intranasal administration of APR significantly reduced the SARS-CoV-2 RNA copy numbers in nasal swabs of hamsters as compared with the control group at three days after initiation of treatment (Figure 6A). SARS-CoV-2 RNA copy numbers were either absent or significantly reduced in the lungs of animals from the APR-treated group (Figure 6B). Thus, prophylaxis with APR inhibited SARS-CoV-2 replication and spread to the lungs [66].

The effectiveness of APR prevention was carried out in a prospective study among 32 medical workers of the COVID-19 hospital, including medical and nursing staff who constantly worked in the “red zone” for three months of observation [66]. Along with maintenance therapy, medical workers used APR nasal spray with saline (Gordox) twice a day: in the morning, before entering the red zone and in the evening, after leaving the red zone, 400 KIU twice a day, for a total of 800 KIU daily. This study demonstrated that only 2 out of 30 workers (6.7%) were infected with SARS-CoV-2, disease progression was asymptomatic and was confirmed only by serologic tests at six weeks [66]. 

#### 3.3.2. APR for COVID-19 Treatment

The efficacy of intravenous (IV) and inhaled (Inh) APR was studied in a prospective, single-center study including 10 hospitalized patients with moderate COVID-19-associated pneumonia in each cohort (Table 3, cohorts 1 and 2) [306], and cohort 3 [307,308,309,310]. Cohort 1—IV APR (Gordox^®^ 1,000,000 KIU daily, 3 days) and SOC including oral (PO) hydroxychloroquine (HCQ) (200 mg, twice a day, 5 to 6 days); cohort 2—Inh APR (Gordox^®^ 625 KIU four times per day, 2500 KIU/day, 5 days) and SOC including oral (PO) HCQ (200 mg, twice a day, 5 to 6 days; and SOC [306]); cohort 3—SOC including oral (PO) HCQ (200 mg, twice a day, 5 to 6 days).

This clinical trial [306] can be considered as a treatment for COVID-19 with only one antiviral drug, aprotinin, since HCQ was later shown to have no clinical benefit in COVID-19 [307], because in outpatients with mild-to-moderate COVID-19, HCQ did not reduce the risk of hospitalization compared with placebo control [308], had little or no effect on the risk of death, and did not affect the transition to mechanical ventilation [309]. Therefore, no further trials of HCQ or chloroquine are recommended for the treatment of COVID-19 [313]. 

None of the patients treated with aprotinin were transferred to the ICU for mechanical ventilation or noninvasive ventilation, and their hospital stay was short.

The high efficacy of aprotinin in the treatment of hospitalized patients with moderate COVID-19-associated pneumonia was confirmed by a series of independent tests (Table 3): elimination of the SARS-CoV-2 virus after 7.5–9.0 days; normalization of body temperature after 3.0–4.5 days; normalization of the concentration of C-reactive protein (CRP) at 4.0–6.0 days, which indicates the absence of inflammatory processes in the body of patients; and normalization of D-dimer concentration, indicating the absence of SARS-CoV-2 infection, venous thromboembolism (VTE), disseminated intravascular coagulation (DIC), and thrombosis in patients, which is also confirmed by negative PCR test data and normalization of CRP concentration [310].

Note that some authors assume that the decrease in the D-dimer concentration may be associated with the inhibition of plasmin by antiproteases, which can lead to blocking of fibrinolysis, and this may actually exacerbate the symptoms of DIC in severely ill patients [313]. 

At the same time, Ji et al indicate that cleavage of new furin sites in the S protein of the SARS-CoV-2 virus by plasmin and other proteases can increase its infectivity by accelerating penetration, fusion, duplication, and release in respiratory cells. Elevated plasmin (gene) levels are common in COVID-19 patients with underlying medical conditions. Elevated plasmin(ogen) levels may be an independent risk stratifier in patients with COVID-19. Measurements of plasmin(ogen) levels and enzymatic activity may be important biomarkers of disease severity in addition to the resulting D-dimer. The administration of antiproteases to suppress plasmin activity in the respiratory system may prevent or at least reduce the entry of SARS-CoV-2 into respiratory cells and improve the clinical outcome of patients with COVID-19. In this regard, antiproteases targeting plasmin(ogen), according to Ji et al, may be a promising approach to combat COVID-19 [314].

According to Lippi et al [310], there is currently no doubt that the D-dimer score is the basis for the diagnosis and prognosis of venous thromboembolism (VTE) and disseminated intravascular coagulation (DIC); although new evidence also supports its use for predicting the duration of anticoagulant therapy, especially in patients with unprovoked thrombosis.

The range of clinical applications of D-dimer is gradually expanding beyond traditional thrombotic pathologies to diagnose acute aortic dissection, acute intestinal ischemia and cerebral venous thrombosis among others [315], including also the clinical treatment of COVID-19. Recent results suggest that D-dimer is often elevated in patients with SARS-CoV-2 infection (especially in patients with VTE), which predicts clinical severity (up to death) of COVID-19, and also remains elevated more often with COVID-19 patients with clinical consequences after discharge.

The European Society of Cardiology recently published (May 2022) a clinical indication suggesting that serial measurement of D-dimer may be useful in COVID-19, as elevated values of this biomarker may help identify patients at higher risk of developing venous thromboembolism (VTE) or those requiring high-intensity prophylactic anticoagulant therapy [316].

In another recent paper, the International Colloquium on Biomarkers of COVID-19 Thrombosis endorsed the routine measurement of D-dimer to assess disease severity, VTE risk, and predict SARS-CoV-2 infection; although its assessment for guiding anticoagulant therapy is not currently recommended [317].

On the Covid19-preprints.microbe.ru platform, a published unreviewed preprint of a prospective clinical trial of the safety and efficacy of APR in the treatment of hospitalized patients with moderate-to-severe COVID-19 pneumonia has also been published [59]. This study included 23 patients (14 men and 9 women) with an average age of 60.7 ± 8.3 years. Nine patients (39%) were hospitalized based on a positive PCR test, and 14 patients (61%) were hospitalized based on results of computed tomography and clinical symptoms. The study included patients with moderate (70%) and severe (30%) COVID-19. It was demonstrated that APR treatment effectively and expediently prevented the progression of complications associated with COVID-19, including reduction of the manifestations of systemic inflammation. In this regard, all patients were administered IV with APR (Gordox). On the first day, 500,000 KIU Gordox (5 ampoules of 10 mL per 250 ml of saline) was IV injected, and in the following 3–5 days 1,000,000 KIU Gordox (10 ampoules of 10 mL) was injected per day. The drug was found to be safe to use, and no allergic reactions to the drug or clinically significant side effects were observed. Maintaining a sufficient therapeutic concentration of the APR made it possible to reduce systemic inflammation, as well as to neutralize undesirable effects of the cytokine storm and progression of disease complications. According to computed tomography, stabilization was noted in dynamics with a decrease in lung tissues damage. No deaths of COVID-19 patients were recorded during the observation period, and all patients were discharged from the hospital after treatment.

Later, the efficacy of inhaled APR was also confirmed in a randomized phase III treatment of hospitalized mild-to-moderate COVID-19 patients with pneumonia [318,319]. The study included 32 patients who received SOC + placebo (saline for inhalation) and 28 patients who received SOC + APR by inhalation (500 KIU every 6 h for 10 min up to 2000 KIU/day). 

On the fifth day, there was a significant decrease in dyspnea, heart rate, fibrinogen, glucose, creatinine, and C-reactive protein in the aprotinin group. A statistically significant increase was observed for platelets, ALT, potassium, pCO2, bicarbonate, and lactic acid. In the groups receiving placebo and aprotinin, on the fifth day there was an increase in the concentration of D-dimer (ng/ml): in the placebo group—significantly from 464.0 ± 51.5 to 733.0 ± 221.8 (*p* = 0.014), while in the aprotinin group it was not reliable from 529.9 ± 52.6 to 834.8 ± 226.4 (*p* = 0.221) [318].

Treatment was carried out for 11 days or until patient’s discharge from the hospital. It was found that the duration of treatment in the APR group was two days less than in the placebo group (7.7 ± 0.4 days in placebo group in comparison with 5.8 ± 0.4 days in aprotinin group, *p* = 0.002), the duration of hospitalization was five days shorter (12.6 ± 1.4 days in placebo group against 7.5 ± 0.5 days in aprotinin group, *p* = 0.003), and the frequency of discharges was 2.19 times higher (HR: 2.188 [1.182–4.047]; *p* = 0.013) than in the placebo group. In addition, the APR group needed less oxygen therapy and had no adverse reactions or side effects [318].

#### 3.3.3. APR and Antiviral Drug Combinations for Treatment of Viral Infections

##### Combined Treatment with an Antiviral Drug and APR in Mice Infected with IAV and SARS-CoV-2

**Figure 7:**
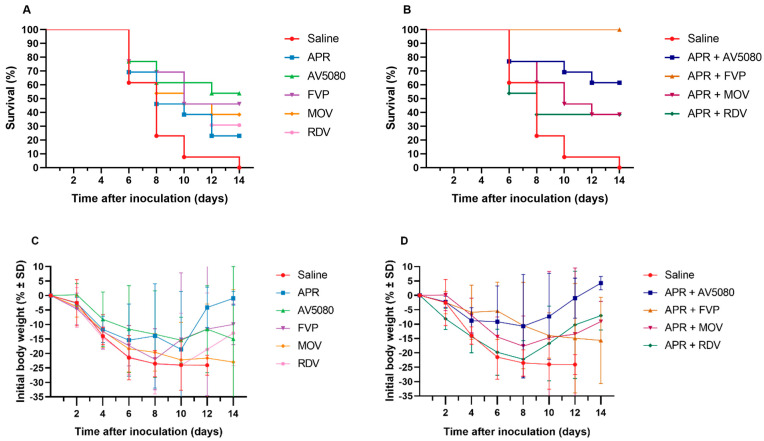
Effect of antiviral treatment on survival (**A**,**B**) and body weight (**C**,**D**) of mice infected with influenza A/California/04/2009 (H1N1)pdm09 virus and treated with hypertonic saline, ARP + AV5080, APR + FVP, APR + MOV, and APR + RDV combinations [68].

Recently, the effectiveness of antiviral treatment with combinations of ARP + AV5080, APR + FVP, APR + MOV, and APR + RDV was studied in a model of influenza pneumonia in mice, and, for comparison, the components included in these combinations were: APR, AV5080, FPV, MOV, and RDV. Anti-influenza activity was studied in a model of influenza pneumonia in mice infected with influenza A/California/04/2009 (H1N1)pdm09 virus (Table 4, Figure 7) [68]. 

The efficacy of APR + AV5080, APR + FVP, APR + MOV, and APR + RDV combinations was compared to a control group of mice that received saline, and the groups of mice that received AV5080, FVP, MOV, RDV, and APR monotherapies (Table 4). Combinations of APR with antiviral drugs were more effective than monotherapies in increasing the average life expectancy of animals, the dynamics of weight loss, and the virus titers in mouse lungs at five days after infection. The most effective were groups consisting of two antivirals with different mechanisms of action: APR + FVP and APR + AV5080 [68].

**Figure 8:**
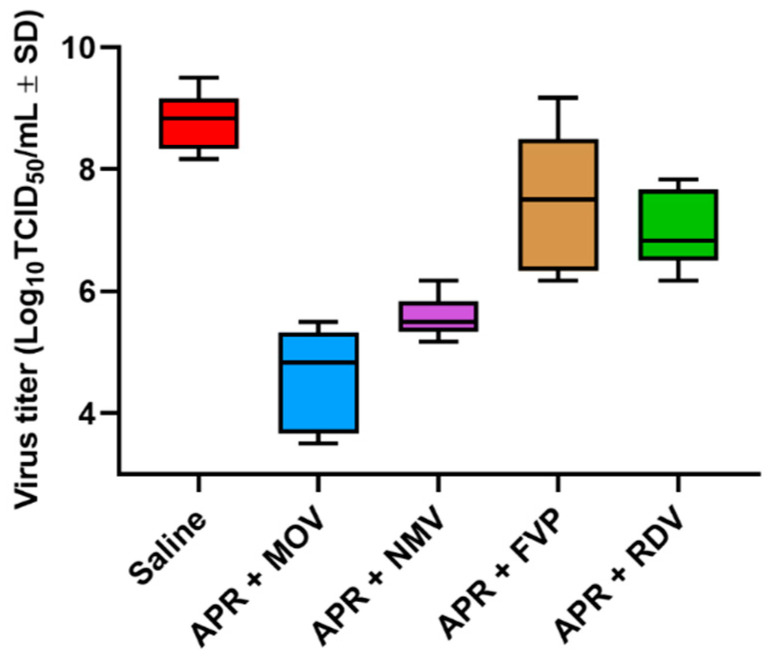
Effect of antiviral treatment on virus titers in the lungs of transgenic mice infected with SARS-CoV-2 and treated with hypertonic saline, ARP + MOV, APR + NMV, APR + FPV, and APR + RDV combinations. Virus titers were determined at four days after infection of transgenic mice with the mouse-adapted SARS-CoV-2 strain [68].

Anti-SARS-CoV-2 activity was studied in transgenic mice [line B6.Cg-Tg(K18-ACE2)2Prlmn/HEMI hemizygous for Tg(K18-ACE2)2Prlmn] infected with SARS-CoV-2. Treatment of mice with APR + MOV, APR + NMV, APR + FVP, and APR + RDV combinations reduced virus titers in the mouse lungs (Figure 8) by 4.2, 3.2, 1.3, and 0.7 orders of magnitude as compared to a group of untreated animals [68]. 

##### Combined Treatment of Hospitalized Patients with Moderate COVID-19-Associated Pneumonia with APR + FVP

The efficacy of APR + FVP combination was studied in a prospective, single-center study including hospitalized patients with moderate COVID-19-associated pneumonia [308]. Patients received a combination of intravenous APR (Gordox^®^ 1,000,000 KIU daily, 5 days), FVP (Avifavir^®^, 2000 mg twice on the first day, then 800 mg twice a day, 10 days), and SOC. Treatment with the combination of APR + FPV was more effective in preventing disease progression in patients hospitalized with COVID-19-associated pneumonia and requiring oxygen therapy than treatment with APR and FPV alone (Table 5).

An analysis of the primary and secondary efficacy points revealed that combination therapy with aprotinin IV APR + Avifavir in association with SOC was beneficial for COVID-19 patients (Table 5). In particular, the median time to SARS-CoV-2 elimination was 3.5 (IQR 3–4) days for cohort 1 and 7.5 (IQR 6–9) days for cohort 2. The median time to CRP normalization was 3.5 (IQR 3–5) days for cohort 1 and 6 (IQR 6–6) days for cohort 2. 

The increased D-dimer levels quickly returned to normal values with a median of 5.0 (IQR 4–5) and 4.5 (IQR 3–6) days for cohorts 1 and 2, respectively.

The median time to normalization of the body temperatures of the patients in cohorts 1 and 2 was 1 (IQR 1–3) and 3 (IQR 2–3) days, respectively. The median time to improve the clinical state by two points was 5 (IQR 5–5) and 11 (IQR 6–11) days for cohorts 1 and 2, respectively.

None of the patients who received combination treatment were transferred to the ICU for mechanical ventilation or noninvasive ventilation, and their duration of hospitalization was short [308].

A retrospective comparison of the above outcomes in hospitalized patients with moderate COVID-19-associated pneumonia with the results of a clinical study of the treatment of similar patients (cohort 3, Table 5) treated with FVP (see [306] Table 1, cohort 4, including avifavir + SOC) shows that combined treatment with APR + FVP is more effective than treatment with APR or FVP alone.

## 4. Conclusions

The studies presented in this review demonstrate the high efficacy of APR for the prevention and treatment of diseases caused by RNA-containing viruses. In this regard, we are convinced that APR revival will put this drug in its rightful place not only in cardiac and noncardiac surgeries, but also in the prevention and treatment of respiratory diseases.

## Figures and Tables

**Table 1:** The inhibition constants Ki for the complexes between APR and the various enzymes [58].

Enzyme-Source-Condition	K_i_
Chemotrypsinogen (bovine), pH 8.0	9.0 nM
CMP-N-Acetylneuraminate lactosylceramide α-2,3-sialyltransferase	74% inhibition at 300.0 nm
Elastase (human leucocytes), pH 8.0	3.5 μM
Kallikrein (pancreatic, porcine), pH 8.0	1.0 nM
Kallikrein (submandibular, porcine), pH 9.0	1.6 nM
Kallikrein (plasma), pH 8	30.0 nM
Kallikrein (plasma), pH 7.8	100.0 nM
Kallikrein (tissue)	0.8 nM; 1.0 nM
Kallikrein (urine, porcine), pH 9.0	1.7 nM
Kallikrein (urine, human), pH 8.0	0.1 nM
Trypsin (bovine), pH 8.0	0.06 pM
Anhydrotrypsin (bovine), pH 8.0	<0.3 pM
Trypsinogen (bovine), pH 8.0	1.8 μM
Chemotrypsin (bovine), pH 8.0	9.0 nM
Chemotrypsin (bovine), pH 7.0	9.0 nM
Plasmin (porcine), pH 7.8	4.0 nM
Plasmin (human), pH 7.8	0.23 nM
Plasminogen activator	8.0 μM; 27.0 μM
Tryptase TL-2	16% inhibition at 10.0 μM

**Table 2:** Antiviral drugs available for influenza treatment.

Viral Protein Target	Mechanism	Inhibitor (Drug)	Virus-IC_50_, nM ^a^
M2 ion channel (IAV)	Interferes with virion and endosomal acidification. Inhibits downstream HA conformation change, endosomal fusion, and release of viral genomes into the cytoplasm	Amantadine (Amantadine: Symmetrel)	J—64.2 µM [139]; IAV M2 proton channel WT (S31)—16.1 µM, IAV M2 proton channel S31N mutant—199.9 µM [140]
		Rimantadine (Rimantadine: Flumadine)	J—67.0 µM [139]; IAV M2 proton channel WT (S31)—10.8 µM, IAV M2 proton channel S31N mutant > 2 mM [140]
Neuraminidase (IAV and IBV)	Blocks NA enzymatic cleavage of host cell sialic acid receptors. Inhibits progeny virus budding	Oseltamivir (Tamiflu)	A—0.58 nM [141], A—1.04 nM [142], B—296.85 ± 8.41 nM, C—0.42 nM, D—172.30 nM [141], H—7.8 nM, I—0.43 nM [142]
		Zanamivir (Relenza)	A—0.38 nM [141]; A—0.35 nM [142]; B—0.44 ± 0.00 nM, C—0.23 nM, D—0.33 nM [141]; H—0.8 nM, I—0.47 nM [142]
		Peramivir (Rapivab/Rapiacta/PeramiFlu)	A—0.05 nM, H—0.18 nM, I—0.07 nM [142]
		Laninamivir (Inavir)	Represented IC_50_s from 0.91 nM (A(H1N1)/Yamagata/83/2006) to 40.5 nM (A(H3N2)/Wisconsin/67/2005) for H1N1 (11 strains), H3N2 (15 strains) and type B viruses (23 strains) are presented [143].
PB1 (IAV, IBV and ICV)	Preferentially incorporated by PB1 into viral RNA. Leads to chain elongation termination and/or lethal mutagenesis	Favipiravir (Avigan)	A—17.05 µM, B—5.07 µM, C—15.54 µM, D—11.36 µM [141] ^b^
PA (IAV and IBV)	Blocks PA endonuclease activity necessary to cleave PB2-bound, capped host mRNAs. Halts viral mRNA transcription	Baloxavir marboxil (Xofluza)	E—0.2 nM, F—0.2 nM, G—2.3 nM [144] ^b^
HA (IVs) [145]	Suppresses virus HA cleavage and limits reproduction of human and avian IVs with a single arginine in the HA cleavage site.	APR (Aerus^TM^)	Represented IC_50_s from 11 nM (A/CA/04/09 (H1N1, 2009 pandemic)) to 110 nM (A/Bris/10/07 (H3N2, oseltamivir-resistant)) for IAV (6 strains) and 39 nM for IBV (B/Seoul/32/11 (Yamagata-like) [145].

^a^ A—A/Brisbane/59/2007; B—A/New Jersey/15/2007; C—A/Denmark/524/2009; D—A/Denmark/528/2009; E—A/California/04/2009 (H1N1)pdm09; F—A/Texas/71/2017 (H3N2); G—B/Brisbane/60/2008 (B/Victoria); H—A(H1N1)pdm09-S247N isolate; I—A(H3N2); J—A/Puerto Rico/8/34 (H1N1). ^b^ BXA activity.

**Table 3:** APR treatment efficacy endpoints in hospitalized patients with moderate COVID-19-associated pneumonia.

	Group 1 IV APR + SOC, n = 10	Group 2 Inh APR + SOC, n = 10	Group 3 SOC *, n = 20
Time until SARS-CoV-2 virus elimination			
Median (IQR), days	7.5 (6–9), *p* = 0.019	9.0 (5–9), *p* = 0.006	9.0 (5.0–9.0)
Time to normal body temperature (normal: <37 °C)			
Median (IQR), days	3.0 (2.0–3.0), *p* = 0.053	4.5 (3.0–5.0)	4.0 (1.0–8.0)
Fever (°C) in patients before treatment	38.3 ± 0.1	38.3 ± 0.3	
Time to normalization of CRP concentration (normal or minor elevation: 3 to 10 mg/L)			
Median (IQR), days	6.0 (6.0–6.0), *p* < 0.001	4.0 (3.0–5.0), *p* < 0.001	14.0 (14.0–14.0)
CRP value in patients before treatment	21.5 (±8.2)	38.9 (±8.1)	
Time to normalization of D-dimer concentration (normal: <253 ng/mL)			
Median (IQR), days	4.5 (3–6)	9 (5–9)	*** nt
D-dimer value in patients before treatment	525.4 ± 175.7	820.1 ± 133.1	nt
Time to improvement in clinical status by 2 points on the Ordinal Scale of Clinical Improvement or discharge from the hospital.			
Median (IQR), days	11.0 (6.0–11.0), *p* < 0.001	6.0 (6.0–6.0), *p* < 0.001	13.0 (11.5–15.5)

* SOC (Standard of Care) might include HCQ or other recommended schemes [311,312]. *** not tested.

**Table 4:** Efficacy of treatment with monotherapies and drugs combinations on morbidity and mortality of mice infected with influenza A/California/04/2009 (H1N1)pdm09 virus.

Drug ^a^	Dose (mg/kg)	Lung Titer (mean ± SD, log_10_TCID_50_/mL) ^b^	Survived, No. (P) ^c^	Mortality (%)	Average Life Expectancy (Days)
Control (saline)	N/A	>7.0	0	100	7.6
APR	50,000 ^d^	4.75 ± 0.43	3 (0.0652)	70	10.7
RDV	5	4.33 ± 0.76	4 (0.0248)	60	10.8
MOV	5	4.17 ± 0.58	5 (0.0077)	50	11.4
FVP	5	2.67 ± 0.29	6 (0.0017)	40	12.4
AV5080	0.25	3.92 ± 0.8	7 (0.0002)	30	13.0
APR + RDV	50,000 ^d^ + 5	3.5 ± 0	5 (0.0077)	50	10.4
APR + MOV	50,000 ^d^ + 5	3.5 ± 0.66	5 (0.0077)	50	11.8
APR + FVP	50,000 ^d^ + 5	2.08 ± 0.14	9	0	16.0
APR + AV5080	50,000 ^d^ + 0.25	2.5 ± 0.87	8 (0.00001)	20	14.0

^a^ Mice were lightly anesthetized and inoculated intranasally with 5 MLD_50_ of mouse-adapted influenza A/California/04/2009 (H1N1)pdm09 virus and treated with APR, RDV, MOV, FVP, or AV5080 monotherapies or APR + RDV, APR + MOV, APR + FVP, or APR + AV5080 combinations. APR was administered intraperitoneally, RDV, MOV, FVP, or AV5080 were administered by oral gavage. Control virus-inoculated mice received sterile saline by oral gavage. ^b^ Virus titer in whole lung from A/California/04/2009 (H1N1)pdm09 virus-inoculated mice (n = 3/group) at five days after infection. ^c^ Out of 10 animals; *p* < 0.05 is statistically significant. ^d^ KIU/kg.

**Table 5:** Combination of APR with FVP for the treatment of patients hospitalized with COVID-19.

	Cohort 1, n = 10 (IV APR + PO FVP + SOC *)	Group 2, n = 10 IV APR + SOC	Cohort 3, n = 40 (PO FVP + SOC) [310]
Time until SARS-CoV-2 virus elimination			
Median (IQR), days	3.5 (3–4)	7.5 (6–9), *p* = 0.019	4.5 (4–9)
Time to normalization of CRP concentration (normal or minor elevation: 3 to 10 mg/L)			
Median (IQR), days	3.5 (3–5)	6.0 (6.0–6.0), *p* < 0.001	14.0 (5.5–14)
CRP value in patients before treatment	37.8 ± 6.7	21.5 ± 8.2	
Time to normalization of D-dimer concentration (normal: <253 ng /mL)			
Median (IQR), days	5.0 (4–5)	4.5 (3–6)	NA **
D-dimer value in patients before treatment	855.5 ±142.5	525.4 ±175.7	NA **
Time to normal body temperature (normal: <37 °C)			
Median (IQR), days	1.0 (1–3)	3.0 (2.0–3.0), *p* = 0.053	2.0 (1–3)
Fever (°C) in patients before treatment	38.5 ± 0.4	38.3 ± 0.1	>38.0
Time to improvement in clinical status by 2 points on the Ordinal Scale of Clinical Improvement or discharge from the hospital.			
Median (IQR), days	5.0 (5–5)	11.0 (6.0–11.0), *p* < 0.001	14.0 (11.5–16)

* SOC—might include HCQ or other recommended schemes [309,310]. ** NT—not tested.

## References

[1] Forgie S., Marrie T.J. (2009). Healthcare-associated atypical pneumonia. Semin. Respir. Crit. Care Med..

[2] CDC (2018). History of the 1918 Influenza Pandemic. https://www.cdc.gov/flu/pandemic-resources/1918-commemoration/1918-pandemic-history.htm.

[3] WHO (2023). Coronavirus Disease (COVID-19) Pandemic. https://www.who.int/europe/emergencies/situations/covid-19.

[4] WHO (2022). 14.9 Million Excess Deaths Associated with the COVID-19 Pandemic in 2020 and 2021. https://www.who.int/news/item/05-05-2022-14.9-million-excess-deaths-were-associated-with-the-covid-19-pandemic-in-2020-and-2021.

[5] Haridy R. Study Estimates Real Global COVID Death Toll is Approaching 20 Million. *Health & Wellbeing*, 10 March 2022. https://newatlas.com/health-wellbeing/global-covid19-excess-death-toll-three-times-higher.

[6] Muoio D. WHO Declares End to COVID-19 Global Health Emergency. *Fierce Healthcare*, 5 May 2023. https://www.fiercehealthcare.com/providers/who-declares-end-covid-19-global-health-emergency.

[7] WHO (2023). Influenza (Seasonal). https://www.who.int/news-room/fact-sheets/detail/influenza-(seasonal).

[8] Kilbourne E.D. (2006). Influenza pandemics of the 20th century. Emerg. Infect. Dis..

[9] Baldo V., Bertoncello C., Cocchio S., Fonzo M., Pillon P., Buja A., Baldovin T. (2016). The new pandemic influenza A/(H1N1)pdm09 virus: Is it really “new”?. J. Prev. Med. Hyg..

[10] CDC (2019). 2009 H1N1 Pandemic (H1N1pdm09 Virus). https://www.cdc.gov/flu/pandemic-resources/2009-h1n1-pandemic.html.

[11] Puryear W., Sawatzki K., Hill N., Foss A., Stone J.J., Doughty L., Walk D., Gilbert K., Murray M., Cox E. (2023). Highly Pathogenic Avian Influenza A(H5N1) Virus Outbreak in New England Seals, United States. Emerg. Infect. Dis..

[12] Thompson W.W., Shay D.K., Weintraub E., Brammer L., Cox N., Anderson L.J., Fukuda K. (2003). Mortality associated with influenza and respiratory syncytial virus in the United States. J. Am. Med. Assoc..

[13] Sharma L., Rebaza A., Dela Cruz C.S. (2019). When “B” becomes “A”: The emerging threat of influenza B virus. Eur. Respir. J..

[14] Bui C.H., Chan R.W., Ng M.M., Cheung M.-C., Ng K.-C., Chan M.P., Chan L.L., Fong J.H., Nicholls J., Peiris J.M. (2019). Tropism of influenza B viruses in human respiratory tract explants and airway organoids. Eur. Respir. J..

[15] CDC (2011). Influenza-Associated Pediatric Deaths—United States, September 2010–August 2011. Morb. Mortal. Wkly. Rep..

[16] Tran D., Vaudry W., Moore D., Bettinger J.A., Halperin S.A., Scheifele D.W., Jadvji T., Lee L., Mersereau T., Members of the Canadian Immunization Monitoring Program Active (2016). Hospitalization for Influenza a Versus B. Pediatrics.

[17] Caini S., Schellevis F., El-Guerche Séblain C., Paget J. (2018). Important changes in the timing of influenza epidemics in the WHO European Region over the past 20 years: Virological surveillance 1996 to 2016. Eurosurveillance.

[18] Caini S., Kusznierz G., Garate V.V., Wangchuk S., Thapa B., de Paula Júnior F.J., Ferreira de Almeida W.A., Njouom R., Fasce R.A., Bustos P. (2019). The epidemiological signature of influenza B virus and its B/Victoria and B/Yamagata lineages in the 21st century. PLoS ONE.

[19] CDC (2020). Human Coronaviruses Types. https://www.cdc.gov/coronavirus/types.html.

[20] King A. (2020). An uncommon cold. New Sci..

[21] van Doremalen N., Bushmaker T., Morris D.H., Holbrook M.G., Gamble A., Williamson B.N., Tamin A., Harcourt J.L., Thornburg N.J., Gerber S.I. (2020). Aerosol and Surface Stability of SARS-CoV-2 as Compared with SARS-CoV-1. N. Engl. J. Med..

[22] Thiel V. (2007). Coronaviruses: Molecular and Cellular Biology.

[23] Zumla A., Hui D.S., Perlman S. (2015). Middle East respiratory syndrome. Lancet.

[24] Ramadan N., Shaib H. (2019). Middle East respiratory syndrome coronavirus (MERS-CoV): A review. Germs.

[25] Killerby M.E., Biggs H.M., Midgley C.M., Gerber S.I., Watson J.T. (2020). Middle East Respiratory Syndrome Coronavirus Transmission. Emerg. Infect. Dis..

[26] ECDC (2023). MERS-CoV Worldwide Overview. https://www.ecdc.europa.eu/en/middle-east-respiratory-syndrome-coronavirus-mers-cov-situation-update.

[27] Liu P., Shi L., Zhang W., He J., Liu C., Zhao C., Kong S.K., Loo J.F.C., Gu D., Hu L. (2017). Prevalence and genetic diversity analysis of human coronaviruses among cross-border children. Virol. J..

[28] Lau S.K.P., Luk H.K.H., Wong A.C.P., Li K.S.M., Zhu L., He Z., Fung J., Chan T.T.Y., Fung K.S.C., Woo P.C.Y. (2020). Possible Bat Origin of Severe Acute Respiratory Syndrome Coronavirus 2. Emerg. Infect. Dis..

[29] Chakrabartty I., Khan M., Mahanta S., Chopra H., Dhawan M., Choudhary O.P., Bibi S., Mohanta Y.K., Emran T.B. (2022). Comparative overview of emerging RNA viruses: Epidemiology, pathogenesis, diagnosis and current treatment. Ann. Med. Surg..

[30] Nolen S.W.H.O. Ends Global Health Emergency Designation for Covid. *The New York Times*, 5 May 2023. https://www.nytimes.com/2023/05/05/health/covid-who-emergency-end.html.

[31] WHO (2023). WHO Coronavirus (COVID-19) Dashboard. https://covid19.who.int.

[32] Mangano D.T. (2007). Mortality Associated with Aprotinin during 5 Years Following Coronary Artery Bypass Graft Surgery. JAMA.

[33] Mangano D.T., Tudor I.C., Dietzel C. (2006). The Risk Associated with Aprotinin in Cardiac Surgery. N. Engl. J. Med..

[34] Karkouti K., Beattie W.S., Dattilo K.M., McCluskey S.A., Ghannam M., Hamdy A., Wijeysundera D.N., Fedorko L., Yau T.M. (2006). A propensity score case-control comparison of aprotinin and tranexamic acid in high-transfusion-risk cardiac surgery. Transfusion.

[35] Fergusson D.A., Hébert P.C., Mazer C.D., Fremes S., MacAdams C., Murkin J.M., Teoh K., Duke P.C., Arellano R., Blajchman M.A. (2008). A Comparison of Aprotinin and Lysine Analogues in High-Risk Cardiac Surgery. N. Engl. J. Med..

[36] Shaw A.D., Stafford-Smith M., White W.D., Phillips-Bute B., Swaminathan M., Milano C., Welsby I.J., Aronson S., Mathew J.P., Peterson E.D. (2008). The Effect of Aprotinin on Outcome after Coronary-Artery Bypass Grafting. N. Engl. J. Med..

[37] Schneeweiss S., Seeger J.D., Landon J., Walker A.M. (2008). Aprotinin during Coronary-Artery Bypass Grafting and Risk of Death. N. Engl. J. Med..

[38] FDA (2006). Cardiovascular and Renal Drugs Advisory Committee; Notice of Meeting. Fed. Regist..

[39] Henry D.A., Carless P.A., Moxey A.J., O’Connell D., Stokes B.J., Fergusson D.A., Ker K. (2011). Anti-fibrinolytic use for minimising perioperative allogeneic blood transfusion. Cochrane Database Syst. Rev..

[40] DeAnda A. (2008). Aprotinin and Cardiac Surgery. J. Thorac. Cardiovasc. Surg..

[41] Furnary A.P., Wu Y., Hiratzka L.F., Grunkemeier G.L., Page U.S. (2007). Aprotinin Does Not Increase the Risk of Renal Failure in Cardiac Surgery Patients. Circulation.

[42] Royston D. (2008). Aprotinin; an economy of truth?. J. Thorac. Cardiovasc. Surg..

[43] Pagano D., Howell N.J., Freemantle N., Cunningham D., Bonser R.S., Graham T.R., Mascaro J., Rooney S.J., Wilson I.C., Cramb R. (2008). Bleeding in cardiac surgery: The use of aprotinin does not affect survival. J. Thorac. Cardiovasc. Surg..

[44] Wood S. (2023). FDA Strengthens Safety Warning on Aprotinin Label. *Medscape*. https://www.medscape.com/viewarticle/549554.

[45] Grunkemeier G.L., Wu Y., Furnary A.P. (2009). What is the Value of a p Value?. Ann. Thorac. Surg..

[46] Beattie W.S., Karkouti K. (2011). The Post-BART Anti-Fibrinolytic Dilemma?. J. Cardiothorac. Vasc. Anesth..

[47] DeAnda A., Spiess B.D. (2012). Aprotinin revisited. J. Thorac. Cardiovasc. Surg..

[48] Tempe D., Hasija S. (2012). Are tranexamic acid and ε-aminocaproic acid adequate substitutes for aprotinin?. Ann. Card. Anaesth..

[49] McMullan V., Alston R.P. (2013). Aprotinin and cardiac surgery: A sorry tale of evidence misused. Br. J. Anaesth..

[50] De Hert S.G. (2023). Aprotinin an Old Kid Back on the Block. Presentation on Theme: “Aprotinin an Old Kid Back on the Block”. https://slideplayer.com/slide/15355651/.

[51] De Hert S., Gill R., Habre W., Lance M., Llau J., Meier J., Pouard P., Samama C.M., van der Linden J., van der Linden P. (2015). Aprotinin: Is it time to reconsider?. Eur. J. Anaesthesiol..

[52] European Medicines Agency European Medicines Agency Recommends Lifting Suspension of Aprotinin. *Press Release*, 17 February 2012. https://www.ema.europa.eu/en/documents/press-release/european-medicines-agency-recommends-lifting-suspension-aprotinin_en.pdf.

[53] FDA (2020). List of Approved NDAs for Biological Products That Were Deemed to Be BLAs on 23 March 2020. *FDA*. https://www.fda.gov/media/119229/download.

[54] Ferraris V.A. (2013). Facts, opinions, and conclusions: Aprotinin brings out all of these. J. Thorac. Cardiovasc. Surg..

[55] Hale C., FIERCE Biotech (2018). Nordic Pharma and Clinigen Sign Global Trasylol Supply Deal. https://www.fiercebiotech.com/cro/nordic-pharma-and-clinigen-sign-global-trasylol-supply-deal.

[56] Huber R., Kukla D., Rühlmann A., Epp O., Formanek H. (1970). The basic trypsin inhibitor of bovine pancreas. Die Nat..

[57] Deisenhofer J., Steigemann W., Fritz H., Tschesche H., Greene L.J., Truscheit E. (1974). The Model of the Basic Pancreatic Trypsin Inhibitor Refined at 1.5 Å resolution. Proteinase Inhibitors.

[58] Fritz H., Wunderer G. (1983). Biochemistry and applications of aprotinin, the kallikrein inhibitor from bovine organs. Arzneim. Forsch..

[59] Ivashchenko A., Svistunov A., Khorobryh T., Loginov V., Karapetian R., Mishchenko N., Poyarkov S., Topr M., Volgin M., Yakubova E. (2020). Aprotinin—A new multi-target drug candidate or “magic shotgun” for the therapy of COVID-19. COVID-19-Preprints.

[60] Ivachtchenko A.V., Lavrovsky Y., Okun I. (2016). AVN-101: A Multi-Target Drug Candidate for the Treatment of CNS Disorders. J. Alzheimer’s Dis..

[61] Saenz-Méndez P., Coumar S.M. (2021). Multi-Target Drugs as Master Keys to Complex Diseases: Inverse Docking Strategies and Opportunities. Molecular Docking for Computer-Aided Drug Design.

[62] Youdim M.B.H., Buccafusco J.J. (2005). CNS Targets for multi-functional drugs in the treatment of Alzheimer’s and Parkinson’s diseases. J. Neural Transm..

[63] Peters D.C., Noble S. (1999). Aprotinin. Drugs.

[64] Levi M., Cromheecke M.E., de Jonge E., Prins M.H., de Mol B.J., Briët E., Büller H.R. (1999). Pharmacological strategies to decrease excessive blood loss in cardiac surgery: A meta-analysis of clinically relevant endpoints. Lancet.

[65] Samama C.M. (2004). Aprotinin and major orthopedic surgery. Eur. Spine J..

[66] Ivashchenko A., Svistunov A., Khorobryh T., Loginov V., Karapetian R., Mishchenko N., Poyarkov S., Volgin M., Yakubova E., Topr M. (2020). Aprotinin—A New Drug Candidate for The Prevention of SARS-CoV-2 (COVID-19). COVID-19-Preprints.

[67] Ivashchenko A., Azarova V., Egorova A., Karapetian R., Kravchenko D., Krivonos N., Loginov V., Poyarkov S., Merkulova E., Rosinkova O. (2020). Aprotinin is a potent multi-target drug for the combination therapy of moderate COVID-19 cases. COVID-19-Preprints.

[68] Ivashchenko A.A., Zagribelnyy B.A., Ivanenkov Y.A., Ivashchenko I.A., Karapetian R.N., Kravchenko D.V., Savchuk N.P., Yakubova E.V., Ivachtchenko A.V. (2022). The Efficacy of Aprotinin Combinations with Selected Antiviral Drugs in Mouse Models of Influenza Pneumonia and Coronavirus Infection Caused by SARS-CoV-2. Molecules.

[69] Mahdy A.M., Webster N.R. (2004). Perioperative systemic haemostatic agents. Br. J. Anaesth..

[70] Mannucci P.M. (1998). Hemostatic Drugs. N. Engl. J. Med..

[71] Didiasova M., Wujak L., Schaefer L., Wygrecka M. (2018). Factor XII in coagulation, inflammation and beyond. Cell. Signal..

[72] DrugBank Aprotinin, DB06692. https://go.drugbank.com/drugs/DB06692.

[73] Ascenzi P., Bocedi A., Bolognesi M., Spallarossa A., Coletta M., Cristofaro R., Menegatti E. (2003). The Bovine Basic Pancreatic Trypsin Inhibitor (Kunitz Inhibitor): A Milestone Protein. Curr. Protein Pept. Sci..

[74] Taylor K.M. (2004). Antiinflammatory Effects of Aprotinin. Transfus. Altern. Transfus. Med..

[75] Tassani P., Augustin N., Barankay A., Braun S.L., Zaccaria F., Richter J.A. (2000). High-dose aprotinin modulates the balance between proinflammatory and anti-inflammatory responses during coronary artery bypass graft surgery. J. Cardiothorac. Vasc. Anesth..

[76] Asimakopoulos G., Taylor K.M., Haskard D.O., Landis R.C. (2000). Inhibition of neutrophil L-selectin shedding: A potential anti-inflammatory effect of aprotinin. Perfusion.

[77] Asimakopoulos G., Thompson R., Nourshargh S., Lidington E.A., Mason J.C., Ratnatunga C.P., Haskard D.O., Taylor K.M., Landis R.C. (2000). An anti-inflammatory property of aprotinin detected at the level of leukocyte extravasation. J. Thorac. Cardiovasc. Surg..

[78] Michalets E., Harris L., Topaz O. (2018). Chapter 44—Antifibrinolytics: Pharmacologic Profile and Clinical Utilization in Cardiovascular Thrombus. Cardiovascular Thrombus.

[79] Wachtfogel Y.T., Kucich U., Hack C.E., Gluszko P., Niewiarowski S., Colman R.W., Edmunds L.H.J. (1993). Aprotinin inhibits the contact, neutrophil, and platelet activation systems during simulated extracorporeal perfusion. J. Thorac. Cardiovasc. Surg..

[80] Hill G.E., Alonso A., Spurzem J.R., Stammers A.H., Robbins R.A. (1995). Aprotinin and methylprednisolone equally blunt cardiopulmonary bypass–induced inflammation in humans. J. Thorac. Cardiovasc. Surg..

[81] Harig F., Feyrer R., Mahmoud F., Blum U., Emde J. (1999). von der Reducing the Post-Pump Syndrome by Using Heparin-Coated Circuits, Steroids, or Aprotinin. Thorac. Cardiovasc. Surg..

[82] Hill G.E., Robbins R.A. (1997). Aprotinin but Not Tranexamic Acid Inhibits Cytokine-Induced Inducible Nitric Oxide Synthase Expression. Anesth. Analg..

[83] Soeparwata R., Hartman A.R., Frerichmann U., Stefano G.B., Scheld H.H., Bilfinger T.V. (1996). Aprotinin^®^ diminishes inflammatory processes. Int. J. Cardiol..

[84] Gilliland H.E., Armstrong M.A., Uprichard S., Clarke G., McMurray T.J. (1999). The effect of aprotinin on interleukin-8 concentration and leukocyte adhesion molecule expression in an isolated cardiopulmonary bypass system. Anaesthesia.

[85] Alonso A., Whitten C.W., Hill G.E. (1999). Pump prime only aprotinin inhibits cardiopulmonary bypass-induced neutrophil CD11b up-regulation. Ann. Thorac. Surg..

[86] Bruda N.L., Hurlbert B.J., Hill G.E. (1998). Aprotinin Reduces Nitric Oxide Production in Vitro and in Vivo in a Dose-Dependent Manner. Clin. Sci..

[87] Rahman A., Üstünda B., Burma O., Özercan I.H., Çekirdekçi A., Bayar M.K. (2000). Does aprotinin reduce lung reperfusion damage after cardiopulmonary bypass?. Eur. J. Cardio-Thorac. Surg..

[88] Goto H., Wells K., Takada A., Kawaoka Y. (2001). Plasminogen-Binding Activity of Neuraminidase Determines the Pathogenicity of Influenza A Virus. J. Virol..

[89] Zhirnov O.P., Ovcharenko A.V., Bukrinskaya A.G. (1984). Suppression of Influenza Virus Replication in Infected Mice by Protease Inhibitors. J. Gen. Virol..

[90] Yang J., Li M., Shen X., Liu S. (2013). Influenza A Virus Entry Inhibitors Targeting the Hemagglutinin. Viruses.

[91] Tse L.V., Marcano V.C., Huang W., Pocwierz M.S., Whittaker G.R. (2013). Plasmin-Mediated Activation of Pandemic H1N1 Influenza Virus Hemagglutinin Is Independent of the Viral Neuraminidase. J. Virol..

[92] De Bruin A.C.M., Funk M., Spronken M.I., Gultyaev A.P., Fouchier R.A.M., Richard M. (2022). Hemagglutinin Subtype Specificity and Mechanisms of Highly Pathogenic Avian Influenza Virus Genesis. Viruses.

[93] Schönfelder K., Breuckmann K., Elsner C., Dittmer U., Fistera D., Herbstreit F., Risse J., Schmidt K., Sutharsan S., Taube C. (2021). Transmembrane serine protease 2 Polymorphisms and Susceptibility to Severe Acute Respiratory Syndrome Coronavirus Type 2 Infection: A German Case-Control Study. Front. Genet..

[94] Lazarowitz S.G., Goldberg A.R., Choppin P.W. (1973). Proteolytic cleavage by plasmin of the HA polypeptide of influenza virus: Host cell activation of serum plasminogen. Virology.

[95] Cunha J.P. (2022). Trasylol. *RxList*. https://www.rxlist.com/trasylol-drug.htm#side_effects.%20https://www.drugs.com/sfx/trasylol-side-effects.html.

[96] Dietrich W., Späth P., Ebell A., Richter J.A. (1997). Prevalence of anaphylactic reactions to aprotinin: Analysis of two hundred forty-eight reexposures to aprotinin in heart operations. J. Thorac. Cardiovasc. Surg..

[97] Beierlein W., Scheule A.M., Dietrich W., Ziemer G. (2005). Forty Years of Clinical Aprotinin Use: A Review of 124 Hypersensitivity Reactions. Ann. Thorac. Surg..

[98] Dietrich W., Ebell A., Busley R., Boulesteix A.-L. (2007). Aprotinin and Anaphylaxis: Analysis of 12,403 Exposures to Aprotinin in Cardiac Surgery. Ann. Thorac. Surg..

[99] Royston D., Chhatwani A. (2006). Safety aspects of aprotinin therapy in cardiac surgery patients. Expert Opin. Drug Saf..

[100] Royston D. (2014). The current place of aprotinin in the management of bleeding. Anaesthesia.

[101] Karkouti K., Wijeysundera D.N., Yau T.M., McCluskey S.A., Tait G., Beattie W.S. (2010). The Risk-Benefit Profile of Aprotinin Versus Tranexamic Acid in Cardiac Surgery. Anesth. Analg..

[102] Influenza A virus. Wikipedia. Last edited on 10 May 2023.

[103] Zhirnov O.P. (2016). Asymmetric structure of the influenza A virus and novel function of the matrix protein M1. Probl. Virusol..

[104] Rosário-Ferreira N., Preto A.J., Melo R., Moreira I.S., Brito R.M.M. (2020). The Central Role of Non-Structural Protein 1 (NS1) in Influenza Biology and Infection. Int. J. Mol. Sci..

[105] Varga Z.T., Palese P. (2011). The influenza A virus protein PB1-F2: Killing two birds with one stone?. Virulence.

[106] Dou D., Revol R., Östbye H., Wang H., Daniels R. (2018). Influenza A Virus Cell Entry, Replication, Virion Assembly and Movement. Front. Immunol..

[107] Borau M.S., Silke Stertz S. (2021). Entry of influenza A virus into host cells—Recent progress and remaining challenges. Curr. Opin. Virol..

[108] Moreira E.A., Yamauchi Y., Matthias P. (2021). How Influenza Virus Uses Host Cell Pathways during Uncoating. Cells.

[109] Zhirnov O.P., Bokova N.O., Isaeva E.I., Vorobeva I.V., Konakova T.E., Malyshev N.A. (2014). Therapeutic effect of aerosol form of aprotinin against Influenza. Epidemiol. Infect. Dis..

[110] Böttcher E., Matrosovich T., Beyerle M., Klenk H.-D., Garten W., Matrosovich M. (2006). Proteolytic Activation of Influenza Viruses by Serine Proteases TMPRSS2 and HAT from Human Airway Epithelium. J. Virol..

[111] Böttcher-Friebertshäuser E., Garten W., Matrosovich M., Klenk H.D. (2014). The Hemagglutinin: A Determinant of Pathogenicity. Influenza Pathogenesis and Control—Volume I.

[112] Wettstein L., Kirchhoff F., Münch J. (2022). The Transmembrane Protease TMPRSS2 as a Therapeutic Target for COVID-19 Treatment. Int. J. Mol. Sci..

[113] Chaipan C., Kobasa D., Bertram S., Glowacka I., Steffen I., Tsegaye T.S., Takeda M., Bugge T.H., Kim S., Park Y. (2009). Proteolytic Activation of the 1918 Influenza Virus Hemagglutinin. J. Virol..

[114] Hamilton B.S., Gludish D.W.J., Whittaker G.R. (2012). Cleavage Activation of the Human-Adapted Influenza Virus Subtypes by Matriptase Reveals both Subtype and Strain Specificities. J. Virol..

[115] Hamilton B.S., Whittaker G.R. (2013). Cleavage Activation of Human-adapted Influenza Virus Subtypes by Kallikrein-related Peptidases 5 and 12. J. Biol. Chem..

[116] Zmora P., Blazejewska P., Moldenhauer A.-S., Welsch K., Nehlmeier I., Wu Q., Schneider H., Pöhlmann S., Bertram S. (2014). DESC1 and MSPL Activate Influenza A Viruses and Emerging Coronaviruses for Host Cell Entry. J. Virol..

[117] Limburg H., Harbig A., Bestle D., Stein D.A., Moulton H.M., Jaeger J., Janga H., Hardes K., Koepke J., Schulte L. (2019). TMPRSS2 Is the Major Activating Protease of Influenza A Virus in Primary Human Airway Cells and Influenza B Virus in Human Type II Pneumocytes. J. Virol..

[118] Bertram S., Glowacka I., Blazejewska P., Soilleux E., Allen P., Danisch S., Steffen I., Choi S.-Y., Park Y., Schneider H. (2010). TMPRSS2 and TMPRSS4 Facilitate Trypsin-Independent Spread of Influenza Virus in Caco-2 Cells. J. Virol..

[119] Böttcher-Friebertshäuser E., Freuer C., Sielaff F., Schmidt S., Eickmann M., Uhlendorff J., Steinmetzer T., Klenk H.-D., Garten W. (2010). Cleavage of Influenza Virus Hemagglutinin by Airway Proteases TMPRSS2 and HAT Differs in Subcellular Localization and Susceptibility to Protease Inhibitors. J. Virol..

[120] Böttcher E., Freuer C., Steinmetzer T., Klenk H.-D., Garten W. (2009). MDCK cells that express proteases TMPRSS2 and HAT provide a cell system to propagate influenza viruses in the absence of trypsin and to study cleavage of HA and its inhibition. Vaccine.

[121] Kido H., Takahashi E., Kimoto T. (2019). Role of host trypsin-type serine proteases and influenza virus-cytokine-trypsin cycle in influenza viral pathogenesis. Pathogenesis-based therapeutic options. Biochimie.

[122] Böttcher-Friebertshäuser E. (2018). Membrane-Anchored Serine Proteases: Host Cell Factors in Proteolytic Activation of Viral Glycoproteins. Activation of Viruses by Host Proteases.

[123] Hatesuer B., Bertram S., Mehnert N., Bahgat M.M., Nelson P.S., Pöhlman S., Schughart K. (2013). Tmprss2 Is Essential for Influenza H1N1 Virus Pathogenesis in Mice. PLoS Pathog..

[124] Tarnow C., Engels G., Arendt A., Schwalm F., Sediri H., Preuss A., Nelson P.S., Garten W., Klenk H.-D., Gabriel G. (2014). TMPRSS2 Is a Host Factor That Is Essential for Pneumotropism and Pathogenicity of H7N9 Influenza A Virus in Mice. J. Virol..

[125] Bestle D., Limburg H., Kruhl D., Harbig A., Stein D.A., Moulton H., Matrosovich M., Abdelwhab E.M., Stech J., Böttcher-Friebertshäuser E. (2021). Hemagglutinins of Avian Influenza Viruses Are Proteolytically Activated by TMPRSS2 in Human and Murine Airway Cells. J. Virol..

[126] Braun E., Sauter D. (2019). Furin-mediated protein processing in infectious diseases and cancer. Clin. Transl. Immunol..

[127] Cavallazzi R., Ramirez J.A. (2018). Influenza and Viral Pneumonia. Clin. Chest Med..

[128] McCullers J.A. (2004). Effect of Antiviral Treatment on the Outcome of Secondary Bacterial Pneumonia after Influenza. J. Infect. Dis..

[129] Morris D.E., Cleary D.W., Clarke S.C. (2017). Secondary Bacterial Infections Associated with Influenza Pandemics. Front. Microbiol..

[130] Javanian M., Barary M., Ghebrehewet S., Koppolu V., Vasigala V., Ebrahimpour S. (2021). A brief review of influenza virus infection. J. Med. Virol..

[131] CDC (2022). Disease Burden of Flu. https://www.cdc.gov/flu/about/burden/index.html.

[132] CDC (2021). Archived: Estimated Influenza Illnesses, Medical Visits, Hospitalizations, and Deaths in the United States—2019–2020 Influenza Season. https://www.cdc.gov/flu/about/burden/2019-2020/archive-09292021.html#:~:text=During%20the%202019-2020%20influenza,405%2C000%20hospitalizations%2C%20and%2022%2C000%20deaths.

[133] Blanton L., Mustaquim D., Alabi N., Kniss K., Kramer N., Budd A., Garg S., Cummings C.N., Fry A.M., Bresee J. (2017). Update: Influenza activity—United States, October 2, 2016-February 4, 2017. Morb. Mortal. Wkly. Rep..

[134] Garg S., Jain S., Dawood F.S., Jhung M., Pérez A., D’Mello T., Reingold A., Gershman K., Meek J., Arnold K.E. (2015). Pneumonia among adults hospitalized with laboratory-confirmed seasonal influenza virus infection—United States, 2005–2008. BMC Infect. Dis..

[135] CDC (2022). Seasonal Flu Vaccine Effectiveness Studies. https://www.cdc.gov/flu/vaccines-work/effectiveness-studies.htm.

[136] Soema P.C., Kompier R., Amorij J.-P., Kersten G.F.A. (2015). Current and next generation influenza vaccines: Formulation and production strategies. Eur. J. Pharm. Biopharm..

[137] Tenforde M.W., Kondor R.J.G., Chung J.R., Zimmerman R.K., Nowalk M.P., Jackson M.L., Jackson L.A., Monto A.S., Martin E.T., Belongia E.A. (2020). Effect of Antigenic Drift on Influenza Vaccine Effectiveness in the United States—2019–2020. Clin. Infect. Dis..

[138] Jones J.C., Yen H.-L., Adams P., Armstrong K., Govorkova E.A. (2023). Influenza antivirals and their role in pandemic preparedness. Antivir. Res..

[139] Sokolova A.S., Yarovaya O.I., Semenova M.D., Shtro A.A., Orshanskaya I.R., Zarubaev V.V., Salakhutdinova N.F. (2017). Synthesis and in vitro study of novel borneol derivatives as potent inhibitors of the influenza A virus. Med. Chem. Commun..

[140] Wang J., Ma C., Wang J., Jo H., Canturk B., Fiorin C., Pinto L.H., Lamb R.A., Michael L., Klein M.L. (2013). Discovery of Novel Dual Inhibitors of the Wild-Type and the Most Prevalent Drug-Resistant Mutant, S31N, of the M2 Proton Channel from Influenza A Virus. J. Med. Chem..

[141] Baranovich T., Wong S.S., Armstrong J., Marjuki H., Webby R.J., Webster R.G., Govorkova E.A. (2013). Govorkova T-705 (favipiravir) induces lethal mutagenesis in influenza A H1N1 viruses in vitro. J. Virol..

[142] Oh D.-E., Milde J., Ham Y., Calderón J.P.R., Wedde M., Dürrwald R., Duwe S.C. (2023). Preparing for the Next Influenza Season: Monitoring the Emergence and Spread of Antiviral Resistance. Infect. Drug Resist..

[143] Vavricka C.J., Li Q., Wu Y., Qi J., Wang M., Liu Y., Gao F., Liu J., Feng E., He J. (2011). Structural and functional analysis of laninamivir and its octanoate prodrug reveals group specific mechanisms for influenza NA inhibition. PLoS Pathog..

[144] Ivashchenko A.A., Mitkin O.D., Jones J.C., Nikitin A.V., Koryakova A.G., Karapetian R.N., Kravchenko D.V., Mochalov S.V., Ryakhovskiy A.A., Aladinskiy V. (2020). Synthesis, inhibitory activity and oral dosing formulation of AV5124, the structural analogue of influenza virus endonuclease inhibitor baloxavir. J. Antimicrob. Chemother..

[145] Song E.-J., Españo E., Shim S.-M., Nam J.-H., Kim J., Lee K., Park S.-K., Lee C.-K., Kim J.-K. (2021). Inhibitory effects of aprotinin on influenza A and B viruses in vitro and in vivo. Sci. Rep..

[146] Oxford J.S., Galbraith A. (1980). Antiviral activity of amantadine: A review of laboratory and clinical data. Pharmacol. Ther..

[147] CDC (2022). CDC Seasonal Influenza Vaccine Effectiveness Studies. 2021–2022. https://www.cdc.gov/flu/vaccines-work/effectiveness-studies.htm.

[148] Loregian A., Mercorelli B., Nannetti G., Compagnin C., Palù G. (2014). Antiviral strategies against influenza virus: Towards new therapeutic approaches. Cell. Mol. Life Sci..

[149] Hussain M., Galvin H., Haw T.Y., Nutsford A., Husain M. (2017). Drug resistance in influenza A virus: The epidemiology and management. Infect. Drug Resist..

[150] Dunning J., Thwaites R.S., Openshaw P.J.M. (2020). Seasonal and pandemic influenza: 100 years of progress, still much to learn. Mucosal Immunol..

[151] Farrukee R., Hurt A.C. (2017). Antiviral Drugs for the Treatment and Prevention of Influenza. Curr. Treat. Options Infect. Dis..

[152] De Clercq E. (2006). Antiviral agents active against influenza A viruses. Nat. Rev. Drug Discov..

[153] Gubareva L.V., Besselaar T.G., Daniels R.S., Fry A., Gregory V., Huang W., Hurt A.C., Jorquera P.A., Lackenby A., Leang S.-K. (2017). Global update on the susceptibility of human influenza viruses to neuraminidase inhibitors, 2015–2016. Antivir. Res..

[154] Lina B., Boucher C., Osterhaus A., Monto A.S., Schutten M., Whitley R.J., Nguyen-Van-Tam J.S. (2018). Five years of monitoring for the emergence of oseltamivir resistance in patients with influenza A infections in the Influenza Resistance Information Study. Influenza Other Respir. Viruses.

[155] CDC Influenza Antiviral Drug Resistance. 25 October 2022. https://www.cdc.gov/flu/treatment/antiviralresistance.htm.

[156] Ivachtchenko A.V., Ivanenkov Y.A., Mitkin O.D., Yamanushkin P.M., Bichko V.V., Shevkun N.A., Karapetian R.N., Leneva I.A., Borisova O.V., Veselov M.S. (2014). Novel oral anti-influenza drug candidate AV5080. J. Antimicrob. Chemother..

[157] The National Institutes of Health Dose Range Study to Evaluate the Efficacy and Safety of AV5080 in Patients with Influenza. *NCT05095545*, 27 October 2021. NCT05095545.

[158] Shiraki K., Daikoku T. (2020). Favipiravir, an anti-influenza drug against life-threatening RNA virus infections. Pharmacol. Ther..

[159] Lai S., Qin Y., Cowling B.J., Ren X., Wardrop N.A., Gilbert M., Tsang T.K., Wu P., Feng L., Jiang H. (2016). Global epidemiology of avian influenza A H5N1 virus infection in humans, 1997–2015: A systematic review of individual case data. Lancet Infect. Dis..

[160] Li Q., Zhou L., Zhou M., Chen Z., Li F., Wu H., Xiang N., Chen E., Tang F., Wang D. (2014). Epidemiology of Human Infections with Avian Influenza A(H7N9) Virus in China. N. Engl. J. Med..

[161] Furuta Y., Takahashi K., Fukuda Y., Kuno M., Kamiyama T., Kozaki K., Nomura N., Egawa H., Minami S., Watanabe Y. (2002). In Vitro and In Vivo Activities of Anti-Influenza Virus Compound T-705. Antimicrob. Agents Chemother..

[162] Furuta Y., Gowen B.B., Takahashi K., Shiraki K., Smee D.F., Barnard D.L. (2013). Favipiravir (T-705), a novel viral RNA polymerase inhibitor. Antivir. Res..

[163] Sleeman K., Mishin V.P., Deyde V.M., Furuta Y., Klimov A.I., Gubareva L.V. (2010). In Vitro Antiviral Activity of Favipiravir (T-705) against Drug-Resistant Influenza and 2009 A(H1N1) Viruses. Antimicrob. Agents Chemother..

[164] Guo Y., Rumschlag-Booms E., Wang J., Xiao H., Yu J., Wang J., Guo L., Gao G.F., Cao Y., Caffrey M. (2009). Analysis of hemagglutinin-mediated entry tropism of H5N1 avian influenza. Virol. J..

[165] Takahashi K., Furuta Y., Fukuda Y., Kuno M., Kamiyama T., Kozaki K., Nomura N., Egawa H., Minami S., Shiraki K. (2003). In Vitro and in Vivo Activities of T-705 and Oseltamivir against Influenza Virus. Antivir. Chem. Chemother..

[166] FujiFIlm The New Drug Application Approval of “AVIGAN® Tablet 200mg” in Japan for the Anti-Influenza Virus Drug. 24 March 2014. https://www.toyama-chemical.co.jp/eng/news/news140324e.html.

[167] Hayden F.G., Lenk R.P., Stonis L., Oldham-Creamer C., Kang L.L., Epstein C. (2022). Favipiravir Treatment of Uncomplicated Influenza in Adults: Results of Two Phase 3, Randomized, Double-Blind, Placebo-Controlled Trials. J. Infect. Dis..

[168] Dufrasne F. (2021). Baloxavir Marboxil: An Original New Drug against Influenza. Pharmaceuticals.

[169] Omoto S., Speranzini V., Hashimoto T., Noshi T., Yamaguchi H., Kawai M., Kawaguchi K., Uehara T., Shishido T., Naito A. (2018). Characterization of influenza virus variants induced by treatment with the endonuclease inhibitor baloxavir marboxil. Sci. Rep..

[170] Kawai M., Tomita K., Akiyama T., Okano A., Miyagawa M. (2016). Substituted Pyridine Derivative and Prodrag Thereof. International Patent.

[171] Mishin V.P., Patel M.C., Chesnokov A., Cruz J.D.L., Nguyen H.T., Lollis L., Hodges E., Jang Y., Barnes J., Uyeki T. (2019). Susceptibility of Influenza A, B, C, and D Viruses to Baloxavir1. Emerg. Infect. Dis..

[172] Govorkova E.A., Takashita E., Daniels R.S., Fujisaki S., Presser L.D., Patel M.C., Huang W., Lackenby A., Nguyen H.T., Pereyaslov D. (2022). Global update on the susceptibilities of human influenza viruses to neuraminidase inhibitors and the cap-dependent endonuclease inhibitor baloxavir, 2018–2020. Antivir. Res..

[173] Uehara T., Hayden F.G., Kawaguchi K., Omoto S., Hurt A.C., Jong M.D.D., Hirotsu N., Sugaya N., Lee N., Baba K. (2019). Treatment-Emergent Influenza Variant Viruses with Reduced Baloxavir Susceptibility: Impact on Clinical and Virologic Outcomes in Uncomplicated Influenza. J. Infect. Dis..

[174] Hayden F.G., Sugaya N., Hirotsu N., Lee N., de Jong M.D., Hurt A.C., Ishida T., Sekino H., Yamada K., Portsmouth S. (2018). Baloxavir Marboxil for Uncomplicated Influenza in Adults and Adolescents. N. Engl. J. Med..

[175] Gubareva L.V., Fry A.M. (2020). Baloxavir and Treatment-Emergent Resistance: Public Health Insights and Next Steps. J. Infect. Dis..

[176] Ivashchenko A.A., Mitkin O.D., Jones J.C., Nikitin A.V., Koryakova A.G., Ryakhovskiy A., Karapetian R.N., Kravchenko D.V., Aladinskiy V., Leneva I.A. (2020). Non-rigid Diarylmethyl Analogs of Baloxavir as Cap-Dependent Endonuclease Inhibitors of Influenza Viruses. J. Med. Chem..

[177] Mitkin O.D., Ivachtchenko A.V., Ivashchenko A.A. (2021). Substituted 3,4,12,12a-tetrahydro-1h-[1,4]oxazino [3,4-c]pyrido[2,1-f][1,2,4]triazine-6,8-dione, Pharmaceutical Composition, and Methods for Preparing and Using Same.

[178] Bertram S., Dijkman R., Habjan M., Heurich A., Gierer S., Glowacka I., Welsch K., Winkler M., Schneider H., Hofmann-Winkler H. (2013). TMPRSS2 Activates the Human Coronavirus 229E for Cathepsin-Independent Host Cell Entry and Is Expressed in Viral Target Cells in the Respiratory Epithelium. J. Virol..

[179] Zhirnov O.P., Matrosovich T.Y., Matrosovich M.N., Klenk H.-D. (2011). Aprotinin, a Protease Inhibitor, Suppresses Proteolytic Activation of Pandemic H1N1v Influenza Virus. Antivir. Chem. Chemother..

[180] Zhirnov O.P., Malyshev N.A. (2014). A new direction in the treatment of influenza and acute respiratory viral infections with aprotinin using a manual propellant metered-dose mini-inhaler. Lechaschiy Vrach.

[181] Zhirnov O.P., Bokova N.O., Isaeva E.I., Vorobieva I.V., Malyshev N.A. (2015). Pathogenetic treatment of influenza patients with aerosolized form of aprotinin, a protease inhibitor. Biol. Prod. Prev. Diagn. Treat..

[182] Day J.R., Landis R.C., Taylor K.M. (2006). Aprotinin and the protease-activated receptor 1 thrombin receptor: Antithrombosis, inflammation, and stroke reduction. Semin. Cardiothorac. Vasc. Anesth..

[183] Landis R.C., Asimakopoulos G., Poullis M., Haskard D.O., Taylor K.M. (2001). The antithrombotic and antiinflammatory mechanisms of action of aprotinin. Ann. Thorac. Surg..

[184] Böttcher-Friebertshäuser E., Klenk H.-D., Garten W. (2013). Activation of influenza viruses by proteases from host cells and bacteria in the human airway epithelium. Pathog. Dis..

[185] Zhirnov O.P., Ovcharenko A.V., Bukrinskaya A.G. (1982). A modified plaque assay method for accurate analysis of infectivity of influenza viruses with uncleaved hemagglutinin. Arch. Virol..

[186] Zhirnov O.P., Ovcharenko A.V., Bukrinskaia A.G. (1981). Usovershenstvovannyĭ metod opredeleniia infektsionnoĭ aktivnosti virusa grippa [Improved method of determining the infectivity of the influenza virus]. Vopr. Virusol..

[187] Zhirnov O.P., Ovcharenko A.V., Bukrinskaya A.G. (1985). Myxovirus Replication in Chicken Embryos Can Be Suppressed by Aprotinin Due to the Blockage of Viral Glycoprotein Cleavage. J. Gen. Virol..

[188] Zhirnov O.P., Golyando P.B., Ovcharenko A.V. (1994). Replication of Influenza B virus in chicken embryos is suppressed by exogenous aprotinin. Arch. Virol..

[189] Rosenberg K. (2022). New Data on Paxlovid Reported. AJN Am. J. Nurs..

[190] Zhirnov O.P., Ikizler M.R., Wright P.F. (2002). Cleavage of Influenza A Virus Hemagglutinin in Human Respiratory Epithelium Is Cell Associated and Sensitive to Exogenous Antiproteases. J. Virol..

[191] Goliando P.B., Ovcharenko A.V., Zhirnov O.P. (1992). Inhibition of the reproduction of the influenza B virus by aprotinin. Vopr. Virusol..

[192] Zhirnov O.P., Khanykov A.V. (2013). Aerosolpraparat auf Aprotininbasis zur Behandlung von Virusinfektionen der Atemwege. https://patentimages.storage.googleapis.com/45/c2/06/87905f9e6fec16/EP2594283A1.pdf.

[193] Zhirnov O.P., Klenk H.D., Wright P.F. (2011). Aprotinin and similar protease inhibitors as drugs against influenza. Antivir. Res..

[194] Ovcharenko A.V., Goliando P.B., Zhirnov O.P. (1992). The therapeutic effect of aprotinin inhalations in influenza and paramyxovirus infections in mice. Vopr. Virusol..

[195] Ovcharenko A.V., Zhirnov O.P. (1994). Aprotinin aerosol treatment of influenza and paramyxovirus bronchopneumonia of mice. Antivir. Res..

[196] Zhirnov O.P., Ovcharenko A.V. (1998). Pharmaceutical Aerosol Composition and Application Thereof for Treatment and Prophylaxis of Viral Diseases. https://patents.google.com/patent/US5723439A/zh.

[197] Zhirnov O.P., Khanykov A.V. (2011). Aprotinin Aerosol for Treating Viral Respiratory Infections. https://patents.google.com/patent/RU2425691C1/en.

[198] Zhirnov O.P., Kirzhner L.S., Ovcharenko A.V., Malyshev N.A. (1996). Clinical effectiveness of aprotinin aerosol in influenza and parainfluenza. Vestn. Ross. Akad. Meditsinskikh Nauk..

[199] Al-Amoodi M., Rao K., Rao S., Brewer J.H., Magalski A., Chhatriwalla A.K. (2010). Fulminant Myocarditis Due to H1N1 Influenza. Circ. Hear. Fail..

[200] Ukimura A., Satomi H., Ooi Y., Kanzaki Y. (2012). Myocarditis Associated with Influenza a H1N1pdm2009. Influenza Res. Treat..

[201] Pan H.-Y., Yamada H., Chida J., Wang S., Yano M., Yao M., Zhu J., Kido H. (2011). Up-regulation of ectopic trypsins in the myocardium by influenza A virus infection triggers acute myocarditis. Cardiovasc. Res..

[202] Pan H.-Y., Sun H.-M., Xue L.-J., Pan M., Wang Y.-P., Kido H., Zhu J.-H. (2014). Ectopic trypsin in the myocardium promotes dilated cardiomyopathy after influenza A virus infection. Am. J. Physiol. Heart Circ. Physiol..

[203] Stadler K., Masignani V., Eickmann M., Becker S., Abrignani S., Klenk H.-D., Rappuoli R. (2003). SARS—Beginning to understand a new virus. Nat. Rev. Microbiol..

[204] Shi J.-Y., Pan H.-Y., Liu K., Pan M., Si G.-J. (2020). Expression of ectopic trypsin in atherosclerotic plaques and the effects of aprotinin on plaque stability. Arch. Biochem. Biophys..

[205] Wang M.-Y., Zhao R., Gao L.-J., Gao X.-F., Wang D.-P., Cao J.-M. (2020). SARS-CoV-2: Structure, Biology, and Structure-Based Therapeutics Development. Front. Cell. Infect. Microbiol..

[206] Yang H., Rao Z. (2021). Structural biology of SARS-CoV-2 and implications for therapeutic development. Nat. Rev. Microbiol..

[207] Menéndez J.C. (2022). Approaches to the Potential Therapy of COVID-19: A General Overview from the Medicinal Chemistry Perspective. Molecules.

[208] Wu C., Zheng M., Yang Y., Gu X., Yang K., Li M., Liu Y., Zhang Q., Zhang P., Wang Y. (2020). Furin: A Potential Therapeutic Target for COVID-19. iScience.

[209] Jin Y., Yang H., Ji W., Wu W., Chen S., Zhang W., Duan G. (2020). Virology, Epidemiology, Pathogenesis, and Control of COVID-19. Viruses.

[210] Zumla A., Chan J.F.W., Azhar E.I., Hui D.S.C., Yuen K.-Y. (2016). Coronaviruses—Drug discovery and therapeutic options. Nat. Rev. Drug Discov..

[211] Jackson C.B., Farzan M., Chen B., Choe H. (2021). Mechanisms of SARS-CoV-2 entry into cells. Nat. Rev. Mol. Cell Biol..

[212] Hoffmann M., Kleine-Weber H., Pöhlmann S. (2020). A Multibasic Cleavage Site in the Spike Protein of SARS-CoV-2 Is Essential for Infection of Human Lung Cells. Mol. Cell.

[213] Shang J., Wan Y., Luo C., Ye G., Geng Q., Auerbach A., Li F. (2020). Cell entry mechanisms of SARS-CoV-2. Proc. Natl. Acad. Sci. USA.

[214] Coutard B., Valle C., de Lamballerie X., Canard B., Seidah N.G., Decroly E. (2020). The spike glycoprotein of the new coronavirus 2019-nCoV contains a furin-like cleavage site absent in CoV of the same clade. Antivir. Res..

[215] Fehr A.R., Perlman S. (2015). Coronaviruses: An Overview of Their Replication and Pathogenesis. Coronaviruses.

[216] Wu C.-T., Lidsky P.V., Xiao Y., Cheng R., Lee I.T., Nakayama T., Jiang S., He W., Demeter J., Knight M.G. (2023). SARS-CoV-2 replication in airway epithelia requires motile cilia and microvillar reprogramming. Cell.

[217] Duong B.V., Larpruenrudee P., Fang T., Hossain S.I., Saha S.C., Gu Y., Islam M.S. (2022). Is the SARS CoV-2 Omicron Variant Deadlier and More Transmissible Than Delta Variant?. Int. J. Environ. Res. Public Health.

[218] Katella K. (2021). 5 Things to Know About the Delta Variant. *Yale Medicine*. https://www.yalemedicine.org/nMws/5-things-to-know-delta-variant-covid.

[219] Xu R., Wang W., Zhang W. (2022). As the SARS-CoV-2 virus evolves, should Omicron subvariant BA.2 be subjected to quarantine, or should we learn to live with it?. Front. Public Health.

[220] Li Q., Nie J., Wu J., Zhang L., Ding R., Wang H., Zhang Y., Li T., Liu S., Zhang M. (2021). SARS-CoV-2 501Y.V2 variants lack higher infectivity but do have immune escape. Cell.

[221] Garcia-Beltran W.F., Lam E.C., Denis K.S., Nitido A.D., Garcia Z.H., Hauser B.M., Feldman J., Pavlovic M.N., Gregory D.J., Poznansky M.C. (2021). Multiple SARS-CoV-2 variants escape neutralization by vaccine-induced humoral immunity. Cell.

[222] Cao Y., Yisimayi A., Bai Y., Huang W., Li X., Zhang Z., Yuan T., An R., Wang J., Xiao T. (2021). Humoral immune response to circulating SARS-CoV-2 variants elicited by inactivated and RBD-subunit vaccines. Cell Res..

[223] Roberts G.C. What Is Omicron XE Variant and Is There Cause for Concern? *World Economic Forum*, 20 April 2022. https://www.weforum.org/agenda/2022/04/omicron-xe-virologist-variants-covid19/.

[224] Ulloa A.C., Buchan S.A., Daneman N., Brown K.A. (2022). Estimates of SARS-CoV-2 Omicron Variant Severity in Ontario, Canada. JAMA.

[225] Berg S. XBB.1.5 Omicron Subvariant: Questions Patients May Have. *Public Health*, 2 February 2023. https://www.ama-assn.org/delivering-care/public-health/xbb15-omicron-subvariant-questions-patients-may-have.

[226] Martín-Sánchez F.J., Martínez-Sellés M., García J.M.M., Guillén S.M., Rodríguez-Artalejo F., Ruiz-Galiana J., Cantón R., Ramos P.D.L., García-Botella A., García-Lledó A. (2022). Insights for COVID-19 in 2023. Rev. Española Quimioter..

[227] Fan Y., Li X., Zhang L., Wan S., Zhang L., Zhou F. (2022). SARS-CoV-2 Omicron variant: Recent progress and future perspectives. Signal Transduct. Target. Ther..

[228] Pia L., Rowland-Jones S. (2022). Omicron entry route. Nat. Rev. Immunol..

[229] Willett B.J., Grove J., MacLean O.A., Wilkie C., Lorenzo G.D., Furnon W., Cantoni D., Scott S., Logan N., Ashraf S. (2022). SARS-CoV-2 Omicron is an immune escape variant with an altered cell entry pathway. Nat. Microbiol..

[230] Meng B., Abdullahi A., Ferreira I.A.T.M., Goonawardane N., Saito A., Kimura I., Yamasoba D., Gerber P.P., Fatihi S., Rathore S. (2022). Altered TMPRSS2 usage by SARS-CoV-2 Omicron impacts infectivity and fusogenicity. Nature.

[231] Romano M., Ruggiero A., Squeglia F., Maga G., Berisio R. (2020). A Structural View of SARS-CoV-2 RNA Replication Machinery: RNA Synthesis, Proofreading and Final Capping. Cells.

[232] Pozzi C., Vanet A., Francesconi V., Tagliazucchi L., Tassone G., Venturelli A., Spyrakis F., Mazzorana M., Costi M.P., Tonelli M. (2023). Antitarget, Anti-SARS-CoV-2 Leads, Drugs, and the Drug Discovery–Genetics Alliance Perspective. J. Med. Chem..

[233] Essalmani R., Jain J., Susan-Resiga D., Andréo U., Evagelidis A., Derbali R.M., Huynh D.N., Dallaire F., Laporte M., Delpal A. (2022). Distinctive Roles of Furin and TMPRSS2 in SARS-CoV-2 Infectivity. J. Virol..

[234] Chakraborty C., Sharma A.R., Bhattacharya M., Agoramoorthy G., Lee S.-S. (2021). The Drug Repurposing for COVID-19 Clinical Trials Provide Very Effective Therapeutic Combinations: Lessons Learned from Major Clinical Studies. Front. Pharmacol..

[235] Bojkova D., Widera M., Ciesek S., Wass M.N., Michaelis M., Cinatl J. (2022). Reduced interferon antagonism but similar drug sensitivity in Omicron variant compared to Delta variant of SARS-CoV-2 isolates. Cell Res..

[236] Takashita E., Kinoshita N., Yamayoshi S., Sakai-Tagawa Y., Fujisaki S., Ito M., Iwatsuki-Horimoto K., Chiba S., Halfmann P., Nagai H. (2022). Efficacy of Antibodies and Antiviral Drugs against COVID-19 Omicron Variant. N. Engl. J. Med..

[237] Li P., Wang Y., Lavrijsen M., Lamers M.M., de Vries A.C., Rottier R.J., Bruno M.J., Peppelenbosch M.P., Haagmans B.L., Pan Q. (2022). SARS-CoV-2 Omicron variant is highly sensitive to molnupiravir, nirmatrelvir, and the combination. Cell Res..

[238] Zhao L., Zhong W. (2021). Mechanism of action of favipiravir against SARS-CoV-2: Mutagenesis or chain termination?. Innovation.

[239] Kokic G., Hillen H.S., Tegunov D., Dienemann C., Seitz F., Schmitzova J., Farnung L., Siewert A., Höbartner C., Cramer P. (2021). Mechanism of SARS-CoV-2 polymerase stalling by remdesivir. Nat. Commun..

[240] Tian L., Pang Z., Li M., Lou F., An X., Zhu S., Song L., Tong Y., Fan H., Fan J. (2022). Molnupiravir and Its Antiviral Activity against COVID-19. Front. Immunol..

[241] Marzi M., Vakil M.K., Bahmanyar M., Zarenezhad E. (2022). Paxlovid: Mechanism of Action, Synthesis, and In Silico Study. BioMed Res. Int..

[242] McMahon J.H., Lau J.S.Y., Coldham A., Roney J., Hagenauer M., Price S., Bryant M., Garlick J., Paterson A., Lee S.J. (2022). Favipiravir in early symptomatic COVID-19, a randomised placebo-controlled trial. Eclinical Med..

[243] Wang Y., Zhang D., Du G., Du R., Zhao J., Jin Y., Fu S., Gao L., Cheng Z., Lu Q. (2020). Remdesivir in adults with severe COVID-19: A randomised, double-blind, placebo-controlled, multicentre trial. Lancet.

[244] Wilkinson E. No Reduction in Hospitalisation or Death from Covid Treatment Molnupiravir. *Pulse*, 20 October 2022. https://www.pulsetoday.co.uk/news/clinical-areas/respiratory/no-reduction-in-hospitalisation-or-death-from-covid-treatment-molnupiravir.

[245] Sirijatuphat R., Manosuthi W., Niyomnaitham S., Owen A., Copeland K.K., Charoenpong L., Rattanasompattikul M., Mahasirimongkol S., Wichukchinda N., Chokephaibulkit K. (2022). Early treatment of Favipiravir in COVID-19 patients without pneumonia: A multicentre, open-labelled, randomized control study. Emerg. Microbes Infect..

[246] Furuta Y., Komeno T., Nakamura T. (2017). Favipiravir (T-705), a broad spectrum inhibitor of viral RNA polymerase. Proc. Jpn. Acad. Ser. B.

[247] Aleem A., Kothadia J.P. (2023). Remdesivir. StatPearls [Internet]. Treasure Island (FL).

[248] The United States Food and Drug Administration FDA Approves First Treatment for COVID-19. 22 October 2020. https://www.fda.gov/news-events/press-announcements/fda-approves-first-treatment-covid-19.

[249] Bhanot G., DeLisi C. (2020). Predictions for Europe for the COVID-19 pandemic from a SIR model. medRxiv.

[250] (2021). Repurposed Antiviral Drugs for COVID-19—Interim WHO Solidarity Trial Results. N. Engl. J. Med..

[251] Reuters. Fact Check-No Evidence Remdesivir Is “killing” COVID-19 Patients, Contrary to Social Media Posts. *Reuters Fact Check*, August 2022. https://www.reuters.com/article/factcheck-coronavirus-remdesivir-idUSL1N30111J.

[252] Hsu J. (2020). COVID-19: What now for remdesivir?. BMJ.

[253] (2020). WHO Guideline Development Group Advises against the Use of Remdesivir for COVID-19. *BMJ*. https://www.bmj.com/company/newsroom/who-guideline-development-group-advises-against-use-of-remdesivir-for-covid-19/.

[254] WHO WHO Recommends against the Use of Remdesivir in COVID-19 Patients. 20 November 2020. https://www.who.int/news-room/feature-stories/detail/who-recommends-against-the-use-of-remdesivir-in-covid-19-patients.

[255] Wikipedia Remdesivir. https://en.wikipedia.org/wiki/Remdesivir#Name.

[256] The United States Food and Drug Administration (2020). VEKLURY® (Remdesivir) for Injection, for Intravenous US. https://www.accessdata.fda.gov/drugsatfda_docs/label/2020/214787Orig1s000lbl.pdf.

[257] Hashemian S.M.R., Pourhanifeh M.H., Hamblin M.R., Shahrzad M.K., Mirzaei H. (2022). RdRp inhibitors and COVID-19: Is molnupiravir a good option?. Biomed. Pharmacother..

[258] The Medicines and Healthcare Products Regulatory Agency (MHRA) (2021). First Oral Antiviral for COVID-19, Lagevrio (Molnupiravir), Approved by MHRA. https://www.gov.uk/government/news/first-oral-antiviral-for-covid-19-lagevrio-molnupiravir-approved-by-mhra.

[259] The Medicines and Healthcare Products Regulatory Agency Last Updated 19/10/22—Summary of Product Characteristics for Lagevrio. https://www.gov.uk/government/publications/regulatory-approval-of-lagevrio-molnupiravir/summary-of-product-characteristics-for-lagevrio.

[260] Masyeni S., Iqhrammullah M., Frediansyah A., Nainu F., Tallei T., Emran T.B., Ophinni Y., Dhama K., Harapan H. (2022). Molnupiravir: A lethal mutagenic drug against rapidly mutating severe acute respiratory syndrome coronavirus 2—A narrative review. J. Med. Virol..

[261] Jayk Bernal A., Gomes da Silva M.M., Musungaie D.B., Kovalchuk E., Gonzalez A., Delos Reyes V., Martín-Quirós A., Caraco Y., Williams-Diaz A., Brown M.L. (2022). Molnupiravir for Oral Treatment of COVID-19 in Nonhospitalized Patients. N. Engl. J. Med..

[262] Fischer W.A., Eron J.J., Holman W., Cohen M.S., Fang L., Szewczyk L.J., Sheahan T.P., Baric R., Mollan K.R., Wolfe C.R. (2022). A phase 2a clinical trial of molnupiravir in patients with COVID-19 shows accelerated SARS-CoV-2 RNA clearance and elimination of infectious virus. Sci. Transl. Med..

[263] Caraco Y., Crofoot G.E., Moncada P.A., Galustyan A.N., Musungaie D.B., Payne B., Kovalchuk E., Gonzalez A., Brown M.L., Williams-Diaz A. (2022). Phase 2/3 Trial of Molnupiravir for Treatment of COVID-19 in Nonhospitalized Adults. NEJM Evid..

[264] Merck. Merck and Ridgeback’s Investigational Oral Antiviral Molnupiravir Reduced the Risk of Hospitalization or Death by Approximately 50 Percent Compared to Placebo for Patients with Mild or Moderate COVID-19 in Positive Interim Analysis of Phase 3 Study. 1 October 2021. https://www.merck.com/news/merck-and-ridgebacks-investigational-oral-antiviralmolnupiravir-reduced-the-risk-of-hospitalization-or-death-by-approximately50-percent-compared-to-placebo-for-patients-with-mild-or-moderat/.

[265] Arribas J.R., Bhagani S., Lobo S.M., Khaertynova I., Mateu L., Fishchuk R., Park W.Y., Hussein K., Kim S.W., Ghosn J. (2022). Randomized Trial of Molnupiravir or Placebo in Patients Hospitalized with COVID-19. NEJM Evid..

[266] The United States Food and Drug Administration Fact Sheet for Healthcare Providers: Emergency Use Uthorization for Lagevrio™ (Molnupiravir) Capsules. https://www.fda.gov/media/155054/download.

[267] Reliefweb WHO Updates Its Treatment Guidelines to Include Molnupiravir. 3 Mar 2022. https://reliefweb.int/report/world/who-updates-its-treatment-guidelines-include-molnupiravir?gclid=Cj0KCQjwwfiaBhC7ARIsAGvcPe5EAG7zvtT7RuwaHnYm2wGPNqKitVhZ7x1AAEjehV1s_D3UxAk8kZcaAl6cEALw_wcB.

[268] Ahmad B., Batool M., ul Ain Q., Kim M.S., Choi S. (2021). Exploring the Binding Mechanism of PF-07321332 SARS-CoV-2 Protease Inhibitor through Molecular Dynamics and Binding Free Energy Simulations. Int. J. Mol. Sci..

[269] Eurapean Medicines Apency EMA Issues Advice on Use of Paxlovid (PF-07321332 and RTV Treatment of COVID-19: Rolling Review Starts in Parallel. 16 December 2021. https://www.ema.europa.eu/en/news/ema-issues-advice-use-paxlovid-pf-07321332-ritonavir-treatment-covid-19.

[270] DrugBank Nirmatrelvir. DrugBank Accession Number DB16691. https://go.drugbank.com/drugs/DB16691.

[271] GoodRx (2021). Paxlovid (Nirmatrelvir/Ritonavir). https://www.goodrx.com/paxlovid/what-is.

[272] Jeong J.H., Chokkakula S., Min S.C., Kim B.K., Choi W.-S., Oh S., Yun Y.S., Kang D.H., Lee O.-J., Kim E.-G. (2022). Combination therapy with nirmatrelvir and molnupiravir improves the survival of SARS-CoV-2 infected mice. Antivir. Res..

[273] Rosenke K., Lewis M.C., Feldmann F., Bohrnsen E., Schwarz B., Okumura A., Bohler W.F., Callison J., Shaia C., Bosio C.M. (2023). Combined molnupiravir-nirmatrelvir treatment improves the inhibitory effect on SARS-CoV-2 in macaques. JCI Insight.

[274] Hammond J., Leister-Tebbe H., Gardner A., Abreu P., Bao W., Wisemandle W., Baniecki M., Hendrick V.M., Damle B., Simón-Campos A. (2022). Oral Nirmatrelvir for High-Risk, Nonhospitalized Adults with COVID-19. N. Engl. J. Med..

[275] Lee E., Park S., Choi J.-P., Kim M.-K., Yang E., Ham S.Y., Lee S., Lee B., Yang J.-S., Park B.K. (2023). Short-Term Effectiveness of Oral Nirmatrelvir/Ritonavir Against the SARS-CoV-2 Omicron Variant and Culture-Positive Viral Shedding. J. Korean Med. Sci..

[276] Alotaibi M., Ali A., Bakhshwin D., Alatawi Y., Alotaibi S., Alhifany A., Alharthi B., Alharthi N., Alyazidi A., Alharthi Y. (2021). Effectiveness and Safety of Favipiravir Compared to Hydroxychloroquine for Management of COVID-19: A Retrospective Study. Int. J. Gen. Med..

[277] Duyan M., ÖZTURAN İ.U. (2022). Comparing the effects of hydroxychloroquine, favipiravir, and hydroxychloroquine plus favipiravir on survival of geriatric population with COVID-19-related pneumonia: A propensity score-matched analysis. Turk. J. Geriatr..

[278] Shindo Y., Kondoh Y., Kada A., Doi Y., Tomii K., Mukae H., Murata N., Imai R., Okamoto M., Yamano Y. (2021). Phase II Clinical Trial of Combination Therapy with Favipiravir and Methylprednisolone for COVID-19 with Non-Critical Respiratory Failure. Infect. Dis. Ther..

[279] Murohashi K., Hagiwara E., Kitayama T., Yamaya T., Higa K., Sato Y., Otoshi R., Shintani R., Okabayashi H., Ikeda S. (2020). Outcome of early-stage combination treatment with favipiravir and methylprednisolone for severe COVID-19 pneumonia: A report of 11 cases. Respir. Investig..

[280] Lowe D.M., Brown L.-A.K., Chowdhury K., Davey S., Yee P., Ikeji F., Ndoutoumou A., Shah D., Lennon A., Rai A. (2022). Favipiravir, lopinavir-ritonavir, or combination therapy (FLARE): A randomised, double-blind, 2 × 2 factorial placebo-controlled trial of early antiviral therapy in COVID-19. PLOS Med..

[281] Terada J., Fujita R., Kawahara T., Hirasawa Y., Kinoshita T., Takeshita Y., Isaka Y., Kinouchi T., Tajima H., Tada Y. (2022). Favipiravir, camostat, and ciclesonide combination therapy in patients with moderate COVID-19 pneumonia with/without oxygen therapy: An open-label, single-center phase 3 randomized clinical trial. Eclinical Med..

[282] Kalil A.C., Patterson T.F., Mehta A.K., Tomashek K.M., Wolfe C.R., Ghazaryan V., Marconi V.C., Ruiz-Palacios G.M., Hsieh L., Kline S. (2021). Baricitinib plus Remdesivir for Hospitalized Adults with COVID-19. N. Engl. J. Med..

[283] Rosas I.O., Diaz G., Gottlieb R.L., Lobo S.M., Robinson P., Hunter B.D., Cavalcante A.W., Overcash J.S., Hanania N.A., Skarbnik A. (2021). Tocilizumab and remdesivir in hospitalized patients with severe COVID-19 pneumonia: A randomized clinical trial. Intensive Care Med..

[284] Kasgari H.A., Babamahmoodi F., Badabi A.R.D., Davanloo A.A., Moradimajd P., Samaee H. (2020). Combination Therapy with Remdisivir and Tocilizumab for COVID-19: Lessons for Futures Studies. Arch. Clin. Infect. Dis..

[285] Vulturar D.-M., Neag M.A., Vesa  Ștefan C., Maierean A.-D., Gherman D., Buzoianu A.D., Orăsan O.H., Todea D.-A. (2022). Therapeutic Efficacy and Outcomes of Remdesivir versus Remdesivir with Tocilizumab in Severe SARS-CoV-2 Infection. Int. J. Mol. Sci..

[286] ACTIV-3/TICO LY-CoV555 Study Group (2021). Neutralizing Monoclonal Antibody for Hospitalized Patients with COVID-19. N. Engl. J. Med..

[287] Kalil A.C., Mehta A.K., Patterson T.F., Erdmann N., Gomez C.A., Jain M.K., Wolfe C.R., Ruiz-Palacios G.M., Kline S., Pineda J.R. (2021). Efficacy of interferon beta-1a plus remdesivir compared with remdesivir alone in hospitalised adults with COVID-19: A double-blind, randomised, placebo-controlled, phase 3 trial. Lancet Respir. Med..

[288] Fakharian A., Barati S., Mirenayat M., Rezaei M., Haseli S., Torkaman P., Yousefian S., Dastan A., Jamaati H., Dastan F. (2021). Evaluation of adalimumab effects in managing severe cases of COVID-19: A randomized controlled trial. Int. Immunopharmacol..

[289] Ianevski A., Yao R., Zusinaite E., Lello L.S., Wang S., Jo E., Yang J., Ravlo E., Wang W., Lysvand H. (2021). Synergistic Interferon-Alpha-Based Combinations for Treatment of SARS-CoV-2 and Other Viral Infections. Viruses.

[290] Abdelnabi R., Foo C.S., Kaptein S.J.F., Zhang X., Do T.N.D., Langendries L., Vangeel L., Breuer J., Pang J., Williams R. (2021). The combined treatment of Molnupiravir and Favipiravir results in a potentiation of antiviral efficacy in a SARS-CoV-2 hamster infection model. eBioMedicine.

[291] The United States Food and Drug Administration Coronavirus (COVID-19) Update: FDA Authorizes Drug Combination for Treatment of COVID-19. 19 November 2020. https://www.fda.gov/news-events/press-announcements/coronavirus-covid-19-update-fda-authorizes-drug-combination-treatment-covid-19.

[292] Adem K.A., Shanti A., Stefanini C., Lee S. (2020). Inhibition of SARS-CoV-2 Entry into Host Cells Using Small Molecules. Pharmaceuticals.

[293] Hernández-Mitre M.P., Tong S.Y.C., Denholm J.T., Dore G.J., Bowen A.C., Lewin S.R., Venkatesh B., Hills T.E., McQuilten Z., Paterson D.L. (2022). Nafamostat Mesylate for Treatment of COVID-19 in Hospitalised Patients: A Structured, Narrative Review. Clin. Pharmacokinet..

[294] Hoffmann M., Hofmann-Winkler H., Smith J.C., Krüger N., Arora P., Sørensen L.K., Søgaard O.S., Hasselstrøm J.B., Winkler M., Hempel T. (2021). Camostat mesylate inhibits SARS-CoV-2 activation by TMPRSS2-related proteases and its metabolite GBPA exerts antiviral activity. EBioMedicine.

[295] Kim Y.-S., Jeon S.-H., Kim J., Koh J.H., Ra S.W., Kim J.W., Kim Y., Kim C.K., Shin Y.C., Kang B.D. (2023). A Double-Blind, Randomized, Placebo-Controlled, Phase II Clinical Study to Evaluate the Efficacy and Safety of Camostat Mesylate (DWJ1248) in Adult Patients with Mild to Moderate COVID-19. Antimicrob. Agents Chemother..

[296] Chupp G., Spichler-Moffarah A., Søgaard O.S., Esserman D., Dziura J., Danzig L., Chaurasia R., Patra K.P., Salovey A., Nunez A. (2022). A Phase 2 Randomized, Double-Blind, Placebo-controlled Trial of Oral Camostat Mesylate for Early Treatment of COVID-19 Outpatients Showed Shorter Illness Course and Attenuation of Loss of Smell and Taste. medRxiv.

[297] Kinoshita T., Shinoda M., Nishizaki Y., Shiraki K., Hirai Y., Kichikawa Y., Tsushima K., Shinkai M., Komura N., Yoshida K. (2022). A multicenter, double-blind, randomized, parallel-group, placebo-controlled study to evaluate the efficacy and safety of camostat mesilate in patients with COVID-19 (CANDLE study). BMC Med..

[298] Tobback E., Degroote S., Buysse S., Delesie L., Dooren L.V., Vanherrewege S., Barbezange C., Hutse V., Romano M., Thomas I. (2022). Efficacy and safety of camostat mesylate in early COVID-19 disease in an ambulatory setting: A randomized placebo-controlled phase II trial. Int. J. Infect. Dis..

[299] Gunst J.D., Staerke N.B., Pahus M.H., Kristensen L.H., Bodilsen J., Lohse N., Dalgaard L.S., Brønnum D., Fröbert O., Hønge B. (2021). Efficacy of the TMPRSS2 inhibitor camostat mesilate in patients hospitalized with COVID-19-a double-blind randomized controlled trial. EClinical Med..

[300] Inokuchi R., Kuno T., Komiyama J., Uda K., Miyamoto Y., Taniguchi Y., Abe T., Ishimaru M., Adomi M., Tamiya N. (2021). Association between Nafamostat Mesylate and In-Hospital Mortality in Patients with Coronavirus Disease 2019: A Multicenter Observational Study. J. Clin. Med..

[301] Ivashchenko A.V., Ivashchenko A.A., Savchuk N.F., Ivashchenko A.A., Loginov V.G., Topr M. (2020). Anti-SARS-COV-2 Viral Agent Antiprovir. Patent RU 2738885.

[302] Bojkova D., Bechtel M., McLaughlin K.-M., McGreig J.E., Klann K., Bellinghausen C., Rohde G., Jonigk D., Braubach P., Ciesek S. (2020). Aprotinin Inhibits SARS-CoV-2 Replication. Cells.

[303] Ivachtchenko A.V., Ivashchenko A.A., Ivachtchenko A.A., Ivashchenko I.A., Savchuk N.F. (2023). Combined Prevention and Treatment of Patients with Respiratory Diseases Caused by RNA Viral Infections.

[304] U.S. National Library of Medicine. An Open Non-Comparative Study of the Efficacy and Safety of Aprotinin in Patients Hospitalized with COVID-19. *NCT04527133*, 26 August 2020. NCT04527133.

[305] Li C., Li A.W. (2022). Hypertonic Saline and Aprotinin Inhibit Furin and Nasal Protease to Reduce SARS-CoV-2 Specific Furin Site Cleavage Activity. J. Explor. Res. Pharmacol..

[306] Ivashchenko A.A., Azarova V.N., Egorova A.N., Karapetian R.N., Kravchenko D.V., Krivonos N.V., Loginov V.G., Poyarkov S.V., Merkulova E.A., Rosinkova O.S. (2021). Effect of Aprotinin and Avifavir^®^ Combination Therapy for Moderate COVID-19 Patients. Viruses.

[307] Avezum Á., Oliveira G.B.F., Oliveira H., Lucchetta R.C., Pereira V.F.A., Dabarian A.L., Vieira R.D., Silva D.V., Kormann A.P.M., Tognon A.P. (2022). Hydroxychloroquine versus placebo in the treatment of non-hospitalised patients with COVID-19 (COPE—Coalition V): A double-blind, multicentre, randomised, controlled trial. Lancet Reg. Health Am..

[308] Schwartz I.S., Boulware D.R., Lee T.C. (2022). Hydroxychloroquine for COVID19: The curtains close on a comedy of errors. Lancet Reg. Health-Am..

[309] Singh B., Ryan H., Kredo T., Chaplin M., Fletcher T. (2021). Chloroquine or hydroxychloroquine for prevention and treatment of COVID-19. Cochrane Database Syst. Rev..

[310] Lippi G., Muller F., Favaloro E.J. (2023). D-dimer: Old dogmas, new (COVID-19) tricks. Clin. Chem. Lab. Med..

[311] NIH (2020). An Adaptive Study of Favipiravir Compared to Standard of Care in Hospitalized Patients with COVID-19. ClinicalTrials.gov Identifier: NCT04434248. NCT04434248.

[312] Ivashchenko A.A., Dmitriev K.A., Vostokova N.V., Azarova V.N., Blinow A.A., Egorova A.N., Gordeev I.G., Ilin A.P., Karapetian R.N., Kravchenko D.V. (2020). AVIFAVIR for Treatment of Patients with Moderate Coronavirus Disease 2019 (COVID-19): Interim Results of a Phase II/III Multicenter Randomized Clinical Trial. Clin. Infect. Dis..

[313] Luan B., Huynh T., Cheng X., Lan G., Wang H.-R. (2020). Targeting Proteases for Treating COVID-19. J. Proteome Res..

[314] Ji H.-L., Zhao R., Matalon S., Matthay M.A. (2020). Elevated Plasmin(ogen) as a Common Risk Factor for COVID-19 Susceptibility. Physiol. Rev..

[315] Favresse J., Lippi G., Roy P.-M., Chatelain B., Jacqmin H., Cate H.T., Mullier F. (2018). D-dimer: Preanalytical, analytical, postanalytical variables, and clinical applications. Crit. Rev. Clin. Lab. Sci..

[316] Baigent C., Windecker S., Andreini D., Arbelo E., Barbato E., Bartorelli A.L., Baumbach A., Behr E.R., Berti S., Task Force for the Management of COVID-19 of the European Society of Cardiology (2022). European Society of Cardiology guidance for the diagnosis and management of cardiovascular disease during the COVID-19 pandemic: Part 1-epidemiology, pathophysiology, and diagnosis. Cardiovasc. Res..

[317] Gorog D.A., Storey R.F., Gurbel P.A., Tantry U.S., Berger J.S., Chan M.Y., Duerschmied D., Smyth S.S., Parker W.A.E., Ajjan R.A. (2022). Current and novel biomarkers of thrombotic risk in COVID-19: A consensus statement from the international COVID-19 thrombosis biomarkers colloquium. Nat. Rev. Cardiol..

[318] Redondo-Calvo F.J., Padín J.F., Muñoz-Rodríguez J.R., Serrano-Oviedo L., López-Juárez P., Leal M.L.P., Gasca F.J.G., Martínez M.R., Serrano R.P., Cadena A.S. (2022). Aprotinin treatment against SARS-CoV-2: A randomized phase III study to evaluate the safety and efficacy of a pan-protease inhibitor for moderate COVID-19. Eur. J. Clin. Investig..

[319] Redondo-Calvo F.J., Padín J.F., Martínez-Alarcón J., Muñoz-Rodríguez J.R., Serrano-Oviedo L., López-Juárez P., Leal M.L.P., Gasca F.J.G., Martínez M.R., Serrano R.P. (2022). Inhaled aprotinin reduces viral load in mild-to-moderate inpatients with SARS-CoV-2 infection. Eur. J. Clin. Investig..

